# The effect of taxonomic classification by full-length 16S rRNA sequencing with a synthetic long-read technology

**DOI:** 10.1038/s41598-020-80826-9

**Published:** 2021-01-18

**Authors:** Jinuk Jeong, Kyeongeui Yun, Seyoung Mun, Won-Hyong Chung, Song-Yi Choi, Young-do Nam, Mi Young Lim, Chang Pyo Hong, ChanHyeok Park, Yong Ju Ahn, Kyudong Han

**Affiliations:** 1 Department of Nanobiomedical Science, Dankook University, Cheonan, 31116 Republic of Korea; 2 Microbiome Division, Theragen Bio Co., Ltd, Seongnam-si, Gyeonggi-do 13488 Republic of Korea; 3 Center for Bio-Medical Engineering Core Facility, Dankook University, Cheonan, 31116 Republic of Korea; 4 Research Group of Healthcare, Korea Food Research Institute, Wanju, 55365 Republic of Korea; 5 Department of Pathology, School of Medicine, Chungnam National University, Daejeon, 35015 Republic of Korea; 6 Department of Microbiology, College of Science and Technology, Dankook University, Cheonan, 31116 Republic of Korea; 7 Department of Food Biotechnology, Korea University of Science and Technology, Daejeon, 34113 Republic of Korea

**Keywords:** Microbial genetics, Genetics, Sequencing, DNA sequencing, Next-generation sequencing, Bacterial genomics, Metagenomics, Clinical microbiology, Metagenomics, Microbiome, Bacteria, Microbial communities, Microbiology, Microbial genetics, Bacterial genes

## Abstract

Characterizing the microbial communities inhabiting specimens is one of the primary objectives of microbiome studies. A short-read sequencing platform for reading partial regions of the 16S rRNA gene is most commonly used by reducing the cost burden of next-generation sequencing (NGS), but misclassification at the species level due to its length being too short to consider sequence similarity remains a challenge. Loop Genomics recently proposed a new 16S full-length-based synthetic long-read sequencing technology (sFL16S). We compared a 16S full-length-based synthetic long-read (sFL16S) and V3-V4 short-read (V3V4) methods using 24 human GUT microbiota samples. Our comparison analyses of sFL16S and V3V4 sequencing data showed that they were highly similar at all classification resolutions except the species level. At the species level, we confirmed that sFL16S showed better resolutions than V3V4 in analyses of alpha-diversity, relative abundance frequency and identification accuracy. Furthermore, we demonstrated that sFL16S could overcome the microbial misidentification caused by different sequence similarity in each 16S variable region through comparison the identification accuracy of *Bifidobacterium*, *Bacteroides*, and *Alistipes* strains classified from both methods. Therefore, this study suggests that the new sFL16S method is a suitable tool to overcome the weakness of the V3V4 method.

## Introduction

The microbiota is the total microbial complex containing the wide variety of bacterial species and is found everywhere, from humans (e.g., the microbiota inhabiting animal intestines) to natural environments^1^. Recently, studies regarding the human GUT microbiota have been conducted worldwide, and many, such as the human microbiome project (HMP), have shown that the human GUT microbiota are strongly related to the development of various diseases^2,3^. Therefore, characterizing the diversity and composition of the microbial communities inhabiting specimen is one of the primary objectives of current microbial studies^4–6^. There have historically been many challenges regarding the efficient analysis of microbial communities due to the impossibility of identifying those that cannot be cultured, and microbial identification analysis was previously limited to culture-dependent sequencing methods based on Sanger sequencing technology^7,8^. However, with the recent rapid development of the next-generation sequencing (NGS) technology, metagenome sequencing is becoming a powerful approach to understanding the complex microbial communities in the human GUT^9^. In metagenomic sequencing, the nine hypervariable regions (V1–V9) of 16S rRNA gene are frequently used for determining of the bacterial taxonomy such as genera or species in the diverse microbial population^10^.

The Pyrosequencing-based Roche 454 GS-FLX used in early metagenome studies, despite its new paradigm for microbial research, was discontinued due to some issues, including high base-calling errors and sequencing cost differences^11^. Currently, the second-generation sequencing platforms are being utilized for microbial diversity analysis by reading a single or combination of the hypervariable regions (e.g., V1V2, V3V4, V4, and V5V6 regions on the 16S rRNA gene)^12–14^. In general, high-throughput short-read sequencing of the 16S rRNA gene amplicon based on the Illumina MiSeq 2 × 300 bp platform (Illumina, USA) specifically targets the V3–V4 hypervariable region of the nine variable regions. It is widely used for various metagenome studies by reducing the high-cost burden of NGS^15^. However, this short read amplicon-based platform is not only vulnerable to the identification bias due to the potential chimeric sequences produced during PCR amplification for library construction, but also is limited to microbial classification at the genus level according to a commonly used 16S rRNA gene-based microbial taxonomy database. Therefore, the amplicon method of the partial variable region (V3–V4) is limited for strains with high similarity at the species level^16^. Recently, rapid technical improvement in third-generation sequencing platforms, such as PacBio Single-Molecule Long-Read Sequencing (Pacific Biosystems, USA) allow the reading of sequences with an average length of 10–20 kb^17^. Circular consensus sequencing of PacBio allows amplicon sequences to be recovered with excellent quality^18^. However, challenges include the high economic cost of metagenomics analysis, which requires large amounts of samples and specimens.

In microbiome studies, this long-read sequencing technology has led to changes in the analysis of complex microbial communities by solving the identification accuracy problem that occurs when reading the partial hypervariable region of the 16S rRNA gene^19–22^. However, this platform generates read data with lower nucleotide accuracy than the Illumina platform (~ 15% compared to ~ 0.1% for Illumina) due to random base-calling errors that occur during multiple times sequencing of the same region^23^. Interestingly, Loop Genomics recently has developed a new 16S full-length-based synthetic long-read sequencing technology (sFL16S) that enables long-read sequencing by utilizing an existing Illumina short-read sequencer combined with a unique molecule barcoding technology. This sFL16S technology can be applied to reading the whole variable regions of 16S rRNA gene (V1–V9) to identify the microbial communities in metagenome studies. Through the barcoding technology, fragmented short-reads containing the same barcode are assembled into a single full-length 16S rRNA gene using computational linked-de novo assembly. Compared to taxonomy classification using only a limited variable region of the 16S rRNA gene, sFL16S provides high quality base-resolution and accurately classifies species and sometimes potential strain levels by reducing false positives. In addition, this synthetic long-read is reconstructed using the short-reads with high base accuracy (~ 99.9%) generated by the Illumina sequencer to compensate for missed variant calls due to nucleotide accuracy errors in the single-molecule long-read technology (Loop Genomics, USA).

In this study, we benchmarked the general 16S rRNA amplicon (V3–V4) sequencing methods and the new 16S full-length-based synthetic long-read (sFL16S) technology for evaluation of the bacterial classification efficiency according to 16S amplicon regions. The diversity scores were estimated higher for the 16S full-sequence readings than those of the V3–V4 sequence readings. Moreover, we confirmed that the sFL16S technology defined more bacterial taxa at the species level. Additionally, we showed that 16S full-length sequencing had higher taxonomic accuracy than the 16S partial region when aligned with the bacterial 16S dataset of sequences extracted from public databases. Therefore, our comparative metagenomic approach confirmed that reading the full-length 16S rRNA gene sequence could have better classification resolution in the metagenome study. We also demonstrated that the new metagenome sequencing technology was an appropriate tool to overcome the misclassification issue that was difficult to define correct microbial taxonomy at the species level.

## Results

### Experimental workflow and data processing

**Figure 1:**
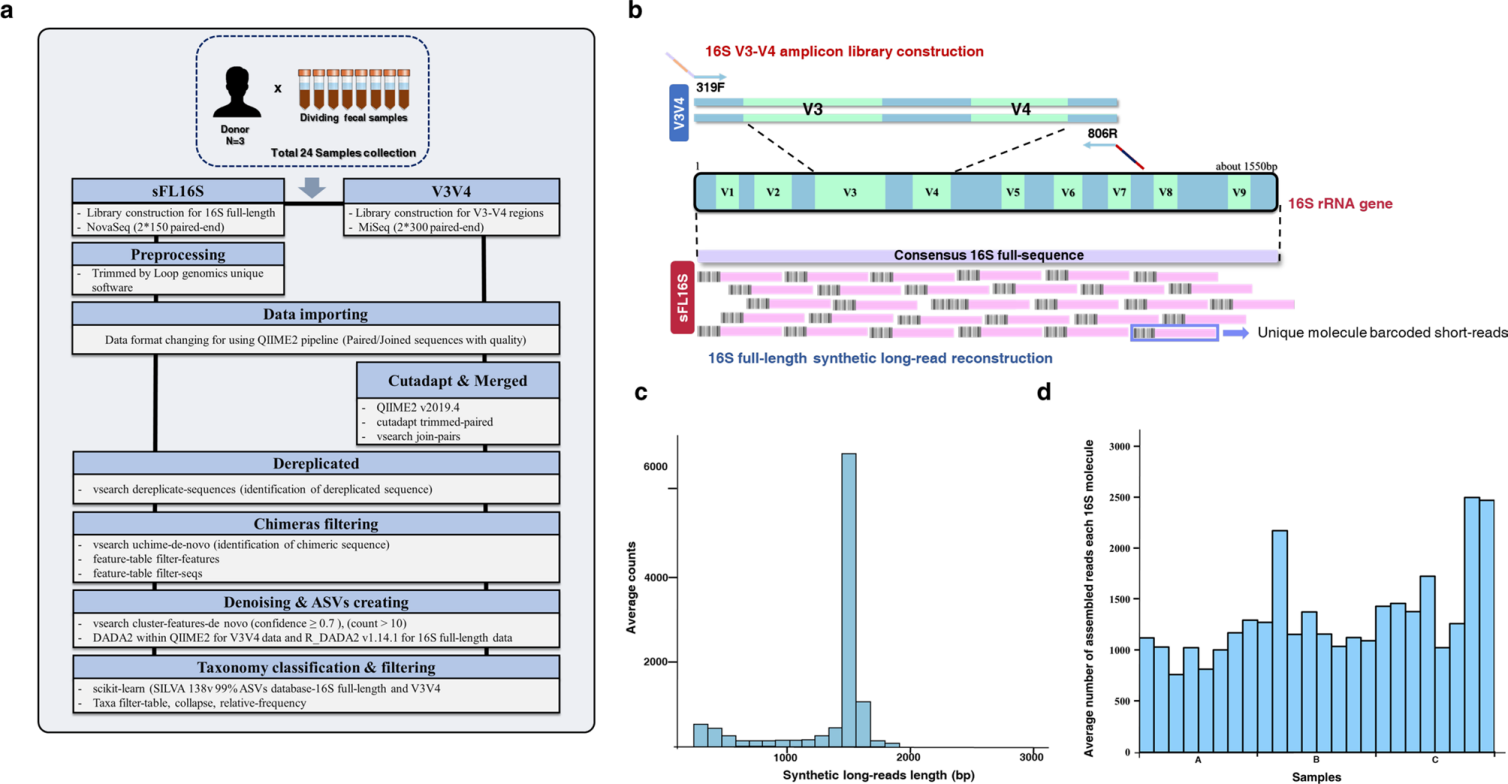
Technical introduction and analysis workflow for a new 16S full-length-based synthetic long-read (sFL16S) technology. Using 24 fecal samples obtained from three healthy adults, we performed metagenomic analysis and aimed to evaluate an efficient method for microbiome screening and bacterial classification by benchmarking two techniques. (**a**) The experimental and analytical workflow of two different approaches in the metagenomic analysis of human GUT microbiota. (**b**) Schematic diagram of the target regions of 16S amplicon library construction for the V3V4 and sFL16S, respectively. For typical 16S amplicon sequencing, the analysis was conducted using the V3V4 variable region. While the short-read targets the V3V4 region, the sFL16S based synthetic long-read targets all variable regions (V1–V9). The high-accurate unique barcoded short-reads (150 PE colored pink), generated from the Illumina Nova-seq were used in the de novo assembly analysis using the UMI tool. (**c**) Density plot for bp length distribution of synthetic long-read sequencing data. (**d**) Bar plot for the average number of assembled short-reads each 16S molecule in each sample we used. The X-axis indicates the octuplicate data for each individual.

We collected human fecal samples from three healthy adults (two men and one woman). To verify the significance of the results, 24 specimens were prepared by dividing each sample into eight identical sampling tubes. We confirmed the integrity (e.g., DNA degradation, concentration, and purity) of the extracted microbial genomic DNA by considering the extraction of high-quality metagenomic DNA may affect its accurate microbial quantification^24^. In the case of the LoopSeq 16S Microbiome SSC 24-Plex kit, it consisted of a multiplex workflow that pools at least 24 samples into a single tube per sequencing run, thus, we performed the 16S V3–V4 amplicon sequencing procedure with the same samples. To compare bacterial classification accuracy of both methods based on V3V4 amplicon short-read sequencing (Illumina V3–V4) and new 16S full-length-based synthetic long-read sequencing (sFL16S; Loop Genomics V1–V9), we successfully constructed each sequencing library using the gDNA samples, and high-throughput metagenome sequencing was performed (Fig. 1a–c). As a result of metagenome sequencing, the average demultiplexed reads and synthetic long-reads count generated by the V3V4 and sFL16S were 75,077 and 36,792, respectively, and the average short-reads count assembled per each 16S molecule of the sFL16S was 1,329 (Fig. 1d; Supplementary Table S1). The amplicon sequence variant (ASV) taxonomy number of the two different 16S metagenomic sequencing methods (V3V4 and sFL16S) that classified with > 70% (default) confidence threshold about the sequence alignment with the SILVA reference database was 623 and 1,041, respectively, and these were filtered to 616 and 1,041 bacteria features, respectively. We estimated that the sFL16S classified ASV taxonomy more than V3V4 amplicon method because the sufficient read-length was used to classify the bacterial taxonomy (Table 1, Supplementary Table S2).

**Table 1:** ASVs' count for microbial community analysis of two different metagenome sequencing method.

Range	Unfiltered count [n]		Bacteria filtered count [n]		Unfiltered count [%]		Bacteria filtered count [%]	
	V34	sFL16S	V34	sFL16S	V34	sFL16S	V34	sFL16S
C^a^ < 0.7	2	0	0	0	0.32	0	0	0
0.7 ≤ C ≤ 0.8	84	106	80	106	13.44	10.18	12.99	10.18
0.8 ≤ C ≤ 0.9	76	114	73	114	12.16	10.95	11.85	10.95
0.9 ≤ C	463	821	463	821	74.08	78.87	75.16	78.87

^a^*C* confidence threshold.

### Alpha-diversity analysis in human GUT microbiota

**Figure 2:**
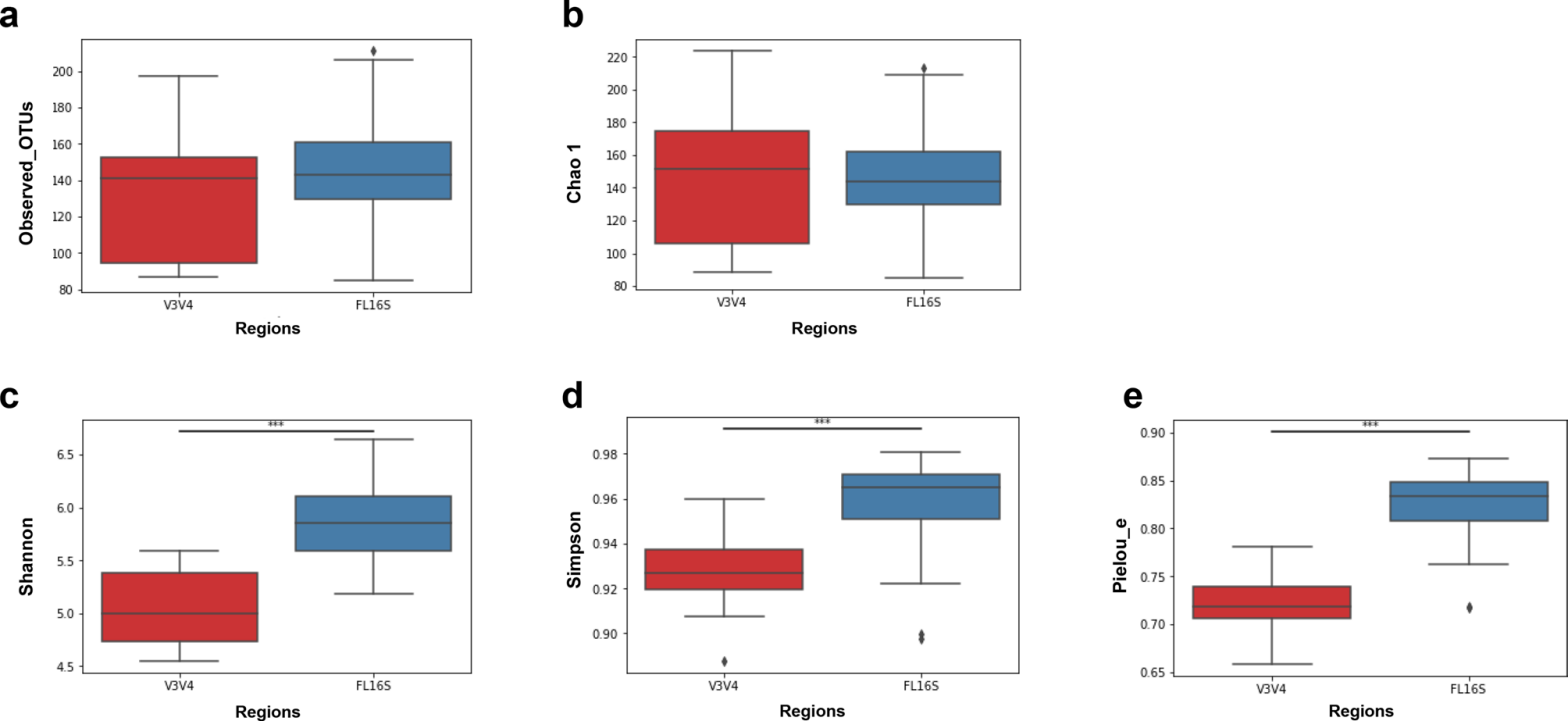
Comparison of average alpha-diversity scores between two different 16S sequencing methods. Box plots show the average alpha-diversity scores of both sequencing methods, which were measured by using (**a**) Observed_OTUs, (**b**) Chao 1, (**c**) Shannon, (**d**) Simpson, and (**e**) Pielou_e indices. Estimation of ASV richness and evenness using the number of observed OTUs (**a**) (Kruskal–Wallis H-test, H = 1.08, P = 0.29), Chao 1 (H = 0.0001, P = 0.99), Shannon diversity index (H = 27.43, P = 1.62*10^–7^), Simpson (H = 17.17, P = 3.4*10^–5^), and Pielou_e (H = 28.74, P = 8.27*10^–8^) are shown. The red and blue boxes indicate average alpha-diversity of V3V4 and sFL16S, respectively. The asterisk (*) represents the *p*-value of the statistical test (*** < 0.0001). Detailed information for this result is in Table 2.

To compare the richness and evenness of the human GUT microbiota classified from the two different methods, we estimated the alpha-diversity that was quantified by Observed_OTUs, Chao1, Shannon, Simpson, and Pielou_e alpha-diversity indices (Fig. 2; Table 2; Supplementary Table S3). In these results, we observed that all estimated alpha-diversity index values calculated were significantly higher in (richness and evenness) the sFL16S method. Considering the result of the Kruskal–Wallis non-parametric test comparing the estimates of alpha-diversity between the two methods (V3V4 and sFL16S) according to the read length, the sFL16S method had a high-diversity score because it could define different bacterial strains with similar sequences. In other words, we confirmed that the number of different reads clustered according to the sequence similarity were fewer sFL16S method than for the V3V4 method. In a previous study, Klemetsen et al. have noted that different variable regions and sequence lengths of the 16S rRNA gene will affect the microbial profile differently by taxonomic rank and phylogenetic group^25^. Therefore, we determined that the differences of sequence similarity on the 16S rRNA variable regions covered according to the read-length affected the diversity value calculation estimating their abundance.

**Table 2:** Alpha-diversity statistics using Kruskal–Wallis test of two different sequencing methods.

Alpha diversity index	Kruskal;H	Kruskal; p	V34_Mean	V34_SD^a^	sFL16S_Mean	sFL16S_SD
Obserbed_OTUS	1.0850	0.2975	132.4583	34.3853	145.7500	33.4901
Chao 1	0.0001	0.9918	146.4551	42.9086	147.5010	35.0318
Shannon	27.4303	1.63E−07	5.0520	0.3343	5.8669	0.3942
Simpson	17.1773	3.4E−05	0.9289	0.0170	0.9565	0.0226
Pielou_e	28.7415	8.27E−08	0.7218	0.0274	0.8208	0.0428

^a^*SD* Standard deviation.

### Comparison of bacterial classification frequency at the taxonomic rank

**Figure 3:**
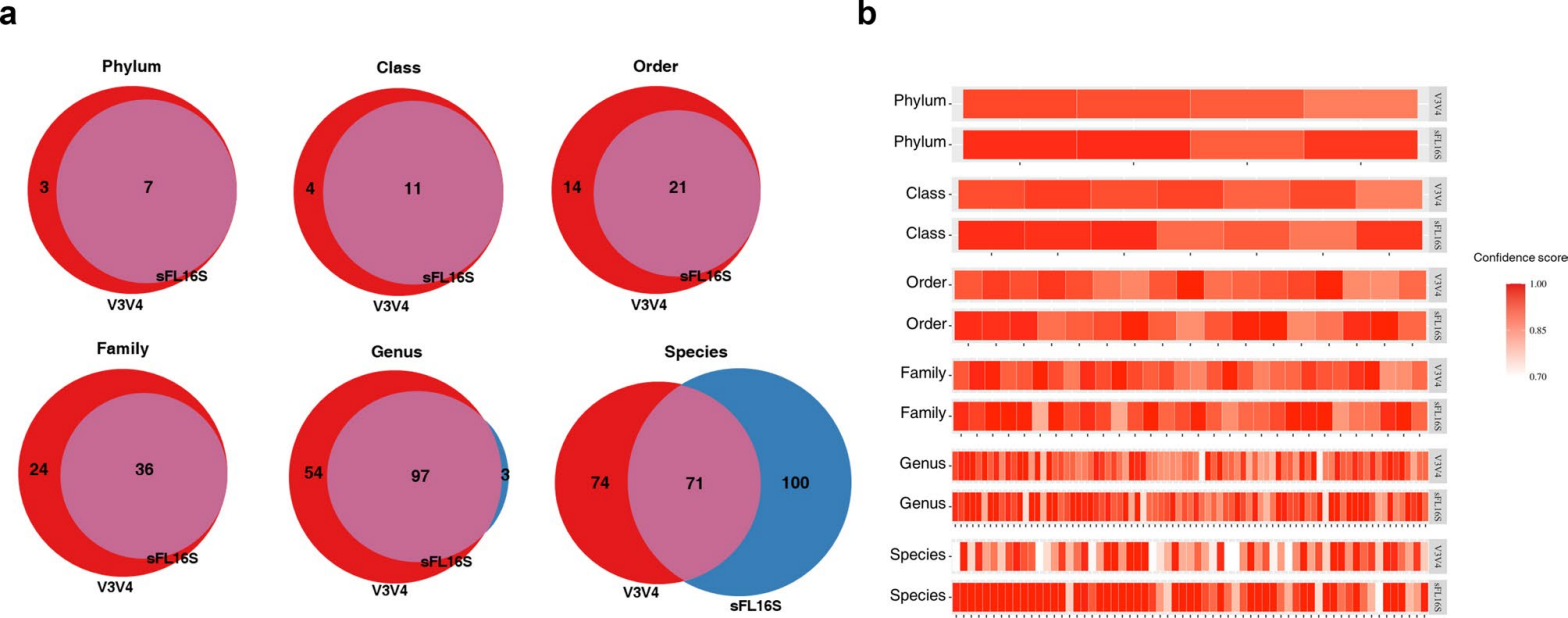
Comparison of bacterial taxonomy classification according to two different 16S amplicon regions. (**a**) The Venn-diagram is divided into two different methods (red: V3-V4 amplicon and blue: sFL16S) and shows the number of a classified bacterial taxon at the phylum to species level. (**b**) The heatmap shows the frequency of the only identified taxa in both methods drawn by each taxonomic rank, in order. The bacterial taxon belonging to the four major phyla (*Bacteroidetes*, *Firmicutes*, *Actinobacteria*, and *Proteobacteria*), to the species, showing the confidence score of each taxon. The darkness of the color is proportional to the confidence score's value, as shown in the color bar.

To support the argument that the length of sequencing read covering the 16S rRNA gene could influence the classification of different bacterial strains, we separated the defined taxon by each taxonomic rank and compared their quantity. The bacterial quantity at each taxonomic rank was visualized by a Venn-diagram which showing the unique and shared taxon between the two methods, and each chart illustrated the distribution of the bacterial taxon which was filtered duplicated taxon name (Fig. 3a). Most of the taxon defined by the sFL16S method from phylum to genus level were included in the classification results by the V3V4 method. Although 54 bacterial taxa were only detected by the V3V4 method at the genus level, the frequency was sufficiently low that their total proportion in the V3V4 genus was only 0.1% (Supplementary Table S4). We assumed that the paired-end V3V4 short-reads had a classification bias down to the genus level compared to the sFL16S synthetic long-reads, which were synthesized through the de novo assembly using short-reads. The reason is that the length of the V3V4 reads was not enough to correctly define the genus corresponding to some reads with unclear sequence accuracy. Therefore, we confirmed that only 100 distinct bacterial species were detected by the sFL16S method compared to the V3V4 method at the species level. The 100 distinct taxa found only in the sFL16S accounted for 24% of its total 171 taxa (relative classification abundance). In contrast, 74 distinct taxa found only in the V3V4 made up 9% of its total 145 taxa. The distinct taxa detected in the V3V4 were, thus, excessively compartmentalized, resulting in a large number of taxa. This results suggested that there is a better advantage to considering 16S full-length to define bacterial taxon at the species level.

Next, we used the classification confidence value in the Supplementary Table S2 to compare the reliability in the number of shared bacterial taxa shown in the Venn-diagram chart at each taxonomic rank (Fig. 3b). For an accurate comparison, bacterial strains belonging to the four major phyla (*Bacteriodetes*, *Firmicutes*, *Actinobacteria*, and *Proteobacteria*) classified in both methods were selected. As a result, we found a significant difference at the species level wherein most shared strains classified by the sFL16S method had nearly 1.00 confidence values (average 0.96) compared to the V3V4 method, which had 0.87 average confidence value. In this respect, these comparison results showed that the 16S full-length sequencing method could classify more bacterial taxa with higher confidence value at the species level than the 16S partial-variable region sequencing method.

### Relative bacterial abundance in human GUT microbiota

**Figure 4:**
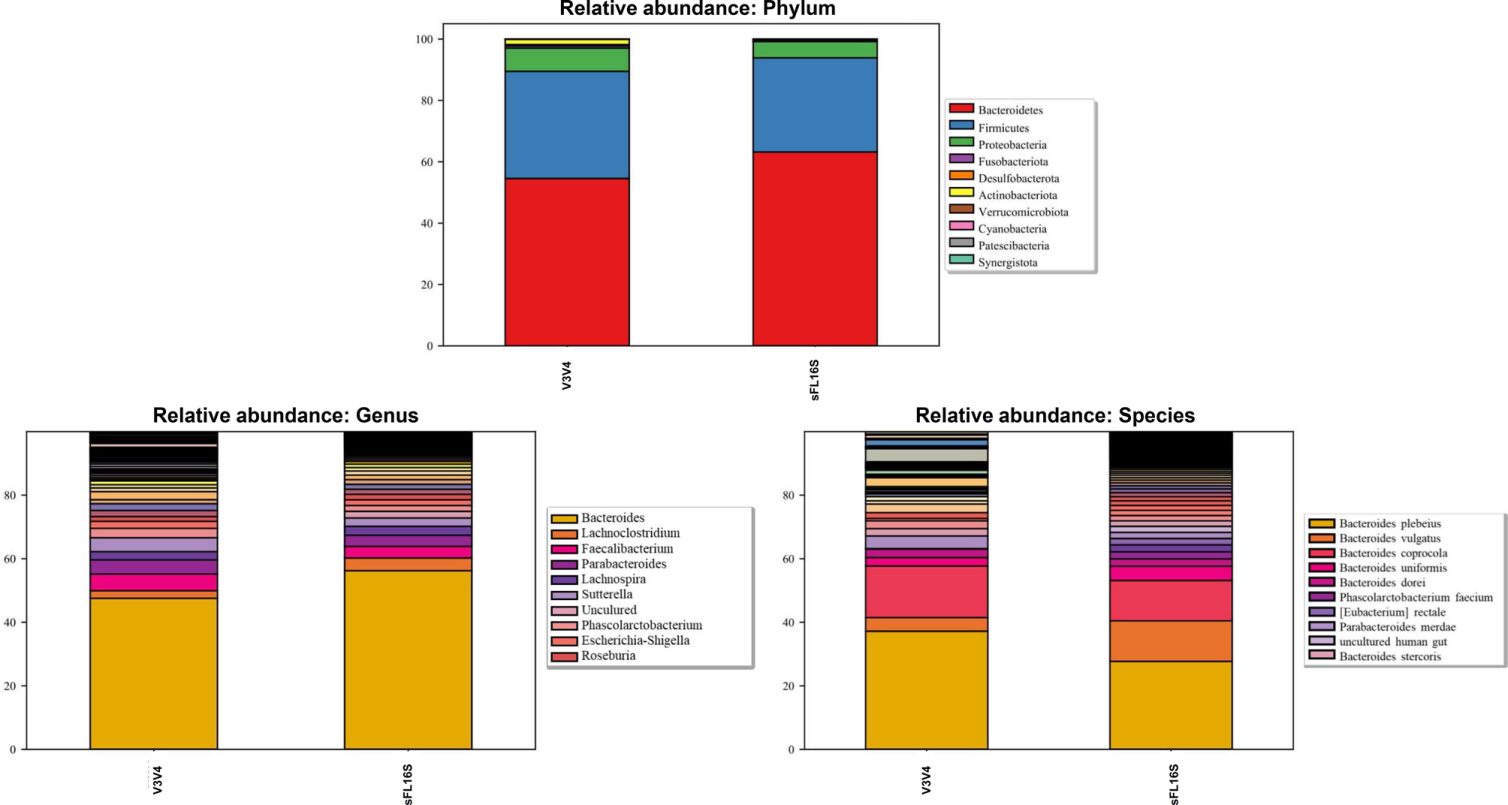
Relative abundance of human GUT microbiota identified from V3V4 and sFL16S methods (phylum, genus, and species). Relative abundance bar plots represent the bacterial composition in human GUT microbiota at the phylum, genus, and species levels, identified from V3V4 and sFL16S methods, respectively. Each legend box shows the top 10 classified bacterial taxa among the whole proportion.

To confirm that the taxonomic profiles were dependent on the 16S read length, relative abundance analysis at each taxonomic rank compared the composition and proportion of the classified bacterial taxa corresponding to data showed in each Venn-diagram chart (Fig. 4; Supplementary Fig. S1; Supplementary Table S4). First, we inspected the phyla composition and proportion of both methods to verify the appropriate classification about the human GUT microbiota. The *Bacteroidetes* and *Firmicutes* were shown to be dominant in both methods. Based on previous studies that the level of GUT microbiota phylum in healthy humans is dominated by the *Bacteroidetes* and *Firmicutes* strains, these results showed to show appropriate bacterial classification in human fecal samples^26–28^. Next, we observed the composition and proportion of the bacterial taxa classified according to all taxonomic rank, determining the bacterial definition between the two different methods. There was no significant difference between the composition and proportion at the phylum to the family level in both methods. Although V3V4 assigned more of 54 distinct bacterial taxa at the genus level, as suggested above, we found that the proportions were very sparse. However, sFL16S showed a more diverse composition of the bacterial strains than V3V4 at the species level. To confirm the incorrect definition of bacterial taxa names at the species level, we compared the proportion of unclear taxa names such as ‘uncultured bacteria’ and ‘Human gut’ within the distinct taxa assigned in each of the two methods. Of the 100 distinct taxa found only in sFL16S, 48 belongs to unclear taxa with a proportion of 34% (relative classification abundance). In contrast, of the 74 distinct taxa found only in the V3V4, 40 belongs to unclear taxa, with a proportion of 98% (Supplementary Fig. S2). Using the same approach, we confirmed that the comparison of inter-individual classification data at the species level also showed the similar patterns (Supplementary Fig. S3). Based on these results, since the sFL16S reads cover the all 16S hypervariable regions and reads almost the entire 16S rRNA sequence, the sFL16S method could distinctly assign the different bacterial taxa with similar sequences at the species level compared to the short-read sequencing method. Furthermore, these results supported a comparison of the classification resolution differences between the two different methods at the species level shown in Fig. 3a. Additionally, we confirmed that the bacterial species defined as indicators related to human health such as *Phascolarctobacterium faecium*, *Roseburia intestinalis*, *Fusobacterium varium*, *Faecalibacterium prausnitzii*, and *Akkermansia muciniphila* were also included in the list of bacterial taxa independently profiled on the sFL16S method^29–33^.

### Verification of misclassification according to 16S read length and sequence similarity

**Figure 5:**
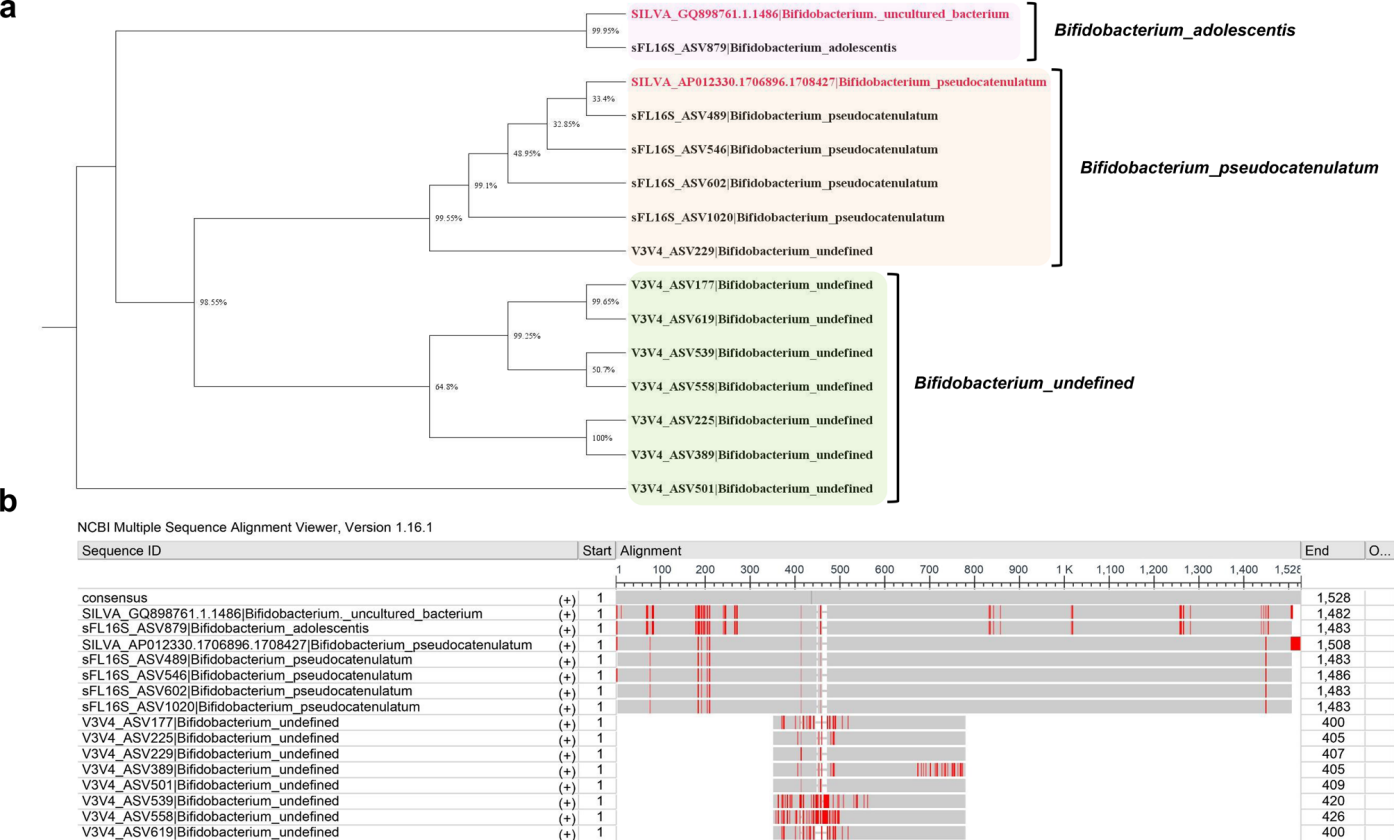
Phylogenetic tree analysis based on distance matrix calculated by multiple sequence alignment (MSA). (**a**) Neighbor-joining phylogenetic tree analysis of the *Bifidobacterium* strains identified in both methods. The 16S reference sequences were obtained from the SILVA 138v non-redundant ribosomal RNA gene database. The pink, orange, and green boxes indicate the clustered ASVs by *Bifidobacterium* species. (**b**) MSA with the reference sequence for the *Bifidobacterium* strains and ASVs identified from V3V4 and sFL16S. The alignment analysis was performed by the IUB multiple alignment matrix with options (transition weight 0.50; delay-divergent cutoff 30%) using MEGA X software. The MSA results visualized using the NCBI Multiple Sequence Alignment Viewer 1.16.1v.

To confirm the premise that 16S read length and sequence similarity could lead to misclassification between different bacterial species, we performed phylogeny analysis of selected bacterial strains distributed in the *Bifidobacterium*, *Bacteroides*, and *Alistipes* genera. The phylogenetic tree was analyzed according to the sequence similarity based on the distance matrix calculated by the multiple sequence alignment (MSA) to the reference sequence in the SILVA 138v database. First, we performed a phylogeny analysis on the *Bifidobacterium* strains, which was difficult to define accurately at the species level due to the high-GC content of genomic DNA and the sequence similarity of some 16S variable regions being from 92 to 99% (Fig. 5)^34–37^. As shown in Fig. 5, the *Bifidobacterium* strains defined by sFL16S showed a high sequence matching rate with the SILVA DB compared to V3V4, and the strains defined in V3V4 were located in an outlier from the reference database. In this phylogenetic tree, SILVA DB connected to sFL16S_ASV879 was assigned as the ‘Uncultured Bacterium’ corresponding to the accession number GQ898761.1 using the BLASTn search by “bit-score”, which was measured by sequence similarity only and did not depend on query sequence length and database size. On the other hand, we confirmed that this SILVA DB was defined the sFL16S_ASV879 as the ‘*Bifidobacterium adolescentis*’ when applying the percent identity calculated for the similarity with the query sequence (Fig. 5a). Additionally, we could also confirm that the eight V3V4 ASVs defined as the ‘*Bifidobacterium_*undefined’ were not accurately assigned at the species level due to the sequence variations caused by errors generating incorrect mapping in the flood of many V3V4 amplicon reads, and the shorter sequence length compared to the full length required to determine the species (Fig. 5b)^1^. Similarly, we performed the phylogenetic analysis on the *Bacteroides* and *Alistipes* phylotypes, which were measured high classification frequencies among the bacterial species classified by the sFL16S (Supplementary Fig. S4). We found no significant differences in these phylogeny analyses compared to Fig. 5 results, except that we defined several bacterial species from the V3V4. Therefore, we verified through the phylogenetic analysis of bacterial species distributed in three genera that the sequence similarity considered according to the 16S amplicon regions could affect the misclassification for bacterial species.

### Cross-check taxonomy profiling using NCBI Bacterial Genome Database

**Figure 6:**
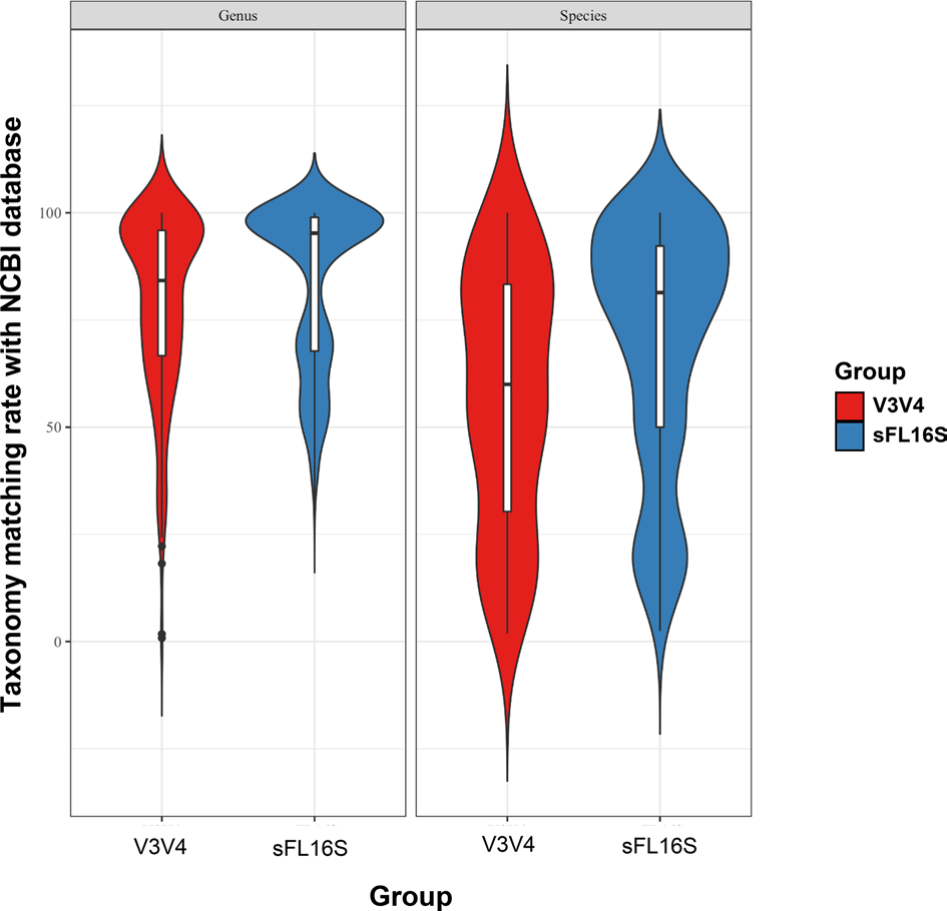
Comparison of taxonomy matching accuracy for V3V4 and sFL16S on NCBI Bacterial Genome Database. Violin plots represent matching rates that result from taxonomy matching analysis in the NCBI Bacterial Genome Database (40 K bacterial and archaeal genomes), according to taxonomic rank (genus and species). Using the NCBI reference 16S sequence data, we conducted the BLAST search to determine the matching rate with the ASV taxonomy data in two different methods. The y-axis indicates the percentiles of the taxonomy matching rate. The violin plots are filled in red (left) and blue (right) for V3V4 and sFL16S data, respectively. The boxes indicate the mean value (black bold horizontal line), percentiles of the taxonomy matching rate distribution. The upper and low whiskers indicate the maximum and the minimum value of the taxonomy matching rate, respectively. The shaded area surrounding the boxes on each side indicate the ASV frequency of the taxonomy matching rate.

To cross-check the taxonomic profiling accuracy of the two different methods, we matched the ASV taxonomy classified from the SILVA 138v database with the extracted 16S rRNA sequences in the NCBI microbial genome sequences (approximately 40 K Genomes) (Fig. 6; Table 3; Supplementary Table S5). As a result, we propose that the ASV taxonomic classifications with a high concordance rate of nearly 100% were more distributed in sFL16S than in V3V4, and that the average concordance rate for the V3V4 ASV taxonomic classification was significantly lower at the species level compared to the genus level. Additionally, we found several cases to understand the mismatch results of some sFL16S ASV taxonomy at the genus and species level. The most discovered case of these reasons was the difference in taxonomy nomenclature form for the same taxon. For example, we confirmed that the strains divided into the *Escherichia* and *Shigella* in the NCBI DB were classified as *Escherichia-Shigella* in the SILVA DB. We also found a case where the *Lacnoclostridium*, named in the SILVA DB, was most often described as *Clostridium* in the NCBI DB. Another reason is that unspecific terms, such as ‘metagenome’ and ‘human_gut’ included in some ASV taxonomy were not found in the NCBI DB. Taken together, we could conclude that the mismatch of some sFL16S ASV taxonomy was not due to misclassification by the base-calling error. In contrast, we presumed that the discrepancy of some V3V4 ASV taxonomy was due to the difficulty in accurately defining different bacterial species with specific-sequences in the other hypervariable regions. Therefore, our findings suggested that the new sFS16S showed high-accuracy for bacterial variant-calling at the species level, as it covered the sequences of all hypervariable regions on the 16S rRNA gene.

**Table 3:** Comparison of the taxon matching rate with NCBI bacterial genome database.

	16S rRNA gene		Genus level		Species level	
*V3V4*						
Matching	419	0.761818	283	0.514545	69	0.125455
Mismatching	131	0.238182	267	0.485455	481	0.874545
*sFL16S*						
Matching	925	0.898931	619	0.601555	348	0.338192
Mismatching	104	0.101069	410	0.398445	681	0.661808

## Discussion

Since the Human Genome Project, projects such as HMP and MetaHIT consortium have been undertaken to identify important associations between microorganisms and diseases^2,38–40^. The projects have revealed that various human diseases are closely related to symbiotic microbes, especially intestinal microflora. In this regard, various studies have been conducted indicating that dysbiosis, which refers to an imbalance of intestinal microflora, is associated with the various human disease^41–43^. Recently, the development of the NGS technologies and reductions in sequencing costs have contributed to the metagenome profiling that could define unculturable microorganisms^44,45^. To interpret the complex community within these human GUT microflora, it is important to correctly define the taxonomy of the microbial strains^46–48^. The 16S rRNA gene, which is well-conserved evolutionarily in the bacterial genome and contains hypervariable regions (V1-V9), is a powerful tool used for microbial taxonomy profiling^49^. However, although the NGS sequencer commonly used for metagenome studies has the advantage of generating NGS data for multiple samples at once, it cannot read the entire 16S rRNA gene sequence (~ 1.5 kb) due to the short-length reading approach (up to 300 bp)^50–52^. To compensate for this problem, 16S amplicons targeting the partially variable regions (V1V2, V3V4, V4, V5V6, etc.) of the 16S rRNA gene are now widely used in the metagenome studies to define various microbial strains^12–14^. Although these amplicon sequences can be applied to confirm the overall microbial composition, some bacterial strains might not be accurately assigned at the species level due to the sequence similarity of the variable regions^53,54^. For this reason, many researchers point out that to accurately define microbial strains with similar taxonomy, sequence similarity should be analyzed by reading the entire 16S rRNA sequence, including all variable regions rather than the partial variable regions^55–57^.

In this study, we are the first to verify the advantages and efficiency of the 16S full-sequence reading in the metagenome study via a novel 16S full-length-based synthetic long-read sequencing technique (sFL16S). This synthetic long-reads has a high sequence accuracy of the reconstructed 16S molecules. The unique molecular barcode short-reads generated by the Illumina sequencer were used for the de novo assembly (Loop Genomics, USA). The unique barcode tag on each short-read indicating the origin of a typical 16S rDNA molecule is identified with the Unique Molecular Identifiers (UMIs) processing tool. In this regard, we assessed that this sFL16S technique is beneficial in classifying the correct microbial taxonomy by distinguishing between sequence similarities. Here, we compared alpha-diversity estimates using each ASV denoising data generated on the general V3V4 method and the new sFL16S method. The relative bacterial beta-diversity between samples was not considered because in-silico analysis estimated the average richness and evenness of the bacterial sequence generated on both methods. As a result of measuring the diversity estimates based on sequencing read length, we confirmed that both the richness and evenness score were calculated higher in the synthetic long-read sequencing method than the V3V4 method. This result could be interpreted that the frequency of estimation for the different strains was high because the number of bacterial sequences clustered according to similarity was small when measuring alpha-diversity in 16S full-sequence reads. When comparing the relative abundance of the bacterial taxonomy classified from two different metagenomic sequencing methods, it was found that the classification frequency at the species level was higher in sFL16S than in V3V4. Although the V3V4 method assigned 54 more unique genera at the genus level, the proportion was only 0.1%. Comparing the relative bacterial composition at the species level between the V3V4 and sFL16S methods, the V3V4 method was found to have a higher frequency of classified taxa containing unspecific terms, such as ‘Uncultured’ and ‘Human gut’ than the sFL16S method. These results indicated that using the V3V4 method, involves reading partial hypervariable regions, it was difficult to accurately assign the bacterial taxa at the species level. Comparing the distribution of confidence score for bacterial species that are commonly classified in both methods, we observed that taxa with score values close to 1.00 were mostly distributed on the sFL16S method. In this respect, we determined that the base-accuracy of synthetic long-reads was similar to that of the reference sequence database. Here, we verified that the difference in sequence similarity according to the amplicon region used in the taxa definition affected bacterial misclassification through phylogeny tree analysis using the MSA method. While the V3V4 ASV taxonomy was placed on an outlier in the SILVA reference taxonomy of the phylogeny tree, the sFL16S was included in the inlier. Through these phylogeny tree analyses, the MSA results suggested that it is possible to accurately define taxa at the species level because the results are derived based on high-similarity scores according to the distance matrix with the reference database. In addition, our classification data were compared to the matching rates in the NCBI Bacterial Genome Database to cross-check the classification accuracy at the genus and species levels of bacterial taxa classified by the two different methods. The bacterial genera and species classified in the sFL16S method had higher matching rates with the NCBI DB than the V3V4 method, and most mismatching indicated in the sFL16S taxon were due to differences in the taxonomy nomenclature between the SILVA and the NCBI DBs. These results showed that the misclassification of the sFL16S at the species level was not just due to the incorrect taxa definitions by base-calling errors. In contrast, the mismatching shown in V3V4 was mostly due to incorrect classifications of different strains. We assumed that the sequence length considered in V3V4 was too short to distinguish between different bacterial species with sequence similarity.

In summary, we applied two different metagenomic sequencing methods to compare the effect on the bacterial profiling efficiency following different 16S hypervariable region readings. We propose that reading the 16S full-sequence could reduce false-positive results on the bacterial classification caused by sequence similarity and might have more advantages in the bacterial diversity analysis. In addition, we suggest that the new sFL16S method is a suitable tool to overcome the weakness of the short-read based-method where it is difficult to define accurate microbial taxonomy at the species level.

## Materials and methods

### Microbial genomic DNA extraction from the human stools

Total microbial metagenomic DNA from each sample was extracted using the QIAamp DNA microbiome kit (Qiagen, Germany) and the experiment was carried out in accordance with the protocol of the DNA extraction kit. The quality of the extracted gDNA was checked using a Bioanalyzer (Agilent 2100, USA) equipment at the Center for Bio-medical Engineering Core Facility (Dankook University, South Korea) and stored at 4 °C until the following process.

### Illumina library construction and sequencing

A total of 24 16S V3–V4 amplicon libraries (eight libraries preps per each participant) were prepared according to the Illumina metagenomic sequencing library construction workflow. The Illumina platform targeted an area containing the V3-V4 hypervariable region of the bacterial 16S rRNA gene. PCR amplification of the target region was performed using the KAPA HiFi Hot Start Ready Mix (2X) (Roche, Mannheim, Germany). For this purpose, a pair of amplicon primers recommended by Illumina were used. After the PCR amplification, the PCR products were purified using the AMPure XP beads (Beckman Coulter, USA). In order to introduce the multiplexing indexes and Illumina sequencing adapters, additional PCR amplification was conducted using the Nextera XT Index Kit (Illumina, USA). The PCR product was then purified once again using the AMPure XP beads. After the library construction, the metagenomic sequencing was performed using the paired-end 2 × 300 bp Illumina MiSeq protocol (Illumina MiSeq, USA)^15^.

### Loop genomics library construction and sequencing

A total of 24 16S full-length based metagenomics sequencing library (eight libraries preps per each participant) were constructed with 10 ng of gDNA extracted from each human stool sample according to the LoopSeq 16S Microbiome SSC 24-Plex kit (Loop Genomics, San Jose, CA, USA) protocol supplied by the Loop Genomics manufacturer. The LoopSeq protocol uses unique molecular barcoding labeling of individual 16S rRNA genes. This unique molecular barcode is evenly distributed throughout the gene and leads to fragmentation of the 16S rRNA gene. The barcoded 16S rRNA gene fragment sequences enable sequencing by short-reads on an Illumina sequencing platform, with subsequent reconstruction of the full-length 16S rRNA genes. Therefore, all hypervariable regions (V1–V9) can be identified and analyzed because the entire 16S rRNA gene is sequenced. The libraries were read on an Illumina NovaSeq 6000 sequencer (Illumina, San Diego, CA, USA), using a paired-end 2 × 150 bp reading system. Coverage was 200–250 million paired-end reads per library of 24 samples. The short-read raw data were collected in real-time on Illumina’s BaseSpace, which generates FASTQ file and then were uploaded to the Loop Genomics unique analytic pipeline^58^.

### Loop genomics 16S full-length preprocessing

The sequencing raw data (2 × 150 bp PE, NovaSeq, Illumina) were transferred to the Loop Genomics unique barcode identifier cloud. It is a data analysis pipeline that is used for the low-quality base trimming, the unique sample barcode demultiplexing, and synthetic long-read reconstruction. The demultiplexing and synthetic long-read reconstruction is a process that enables the de novo assembly to the full-length 16S long-read data after rearranging the short-leads tagged with the same unique barcode.

### 16S metagenomic data analysis

Bacterial 16S rRNA sequencing data of the two different metagenomics sequencing methods were analyzed using QIIME2 next-generation microbiome bioinformatics pipeline for comparative metagenomics study. All raw input data were transformed in the form of QIIME2 artifacts (.qza format), which contain information about the data types and sources for the downstream processing. From raw sequences data, the amplicon sequence variants (ASVs) were obtained using the Divisive Amplicon Denoising Algorithm 2 (DADA2) within QIIME 2 plugin, which detects and corrects amplicon errors and filters out the potential base error and chimeric sequences (Supplementary Table S1)^59,60^. The 16S full-length sequences, which are pre-processed data from Loop Genomics, were filtered, trimming and dereplicating, and then DADA2 (R 1.14.1v) was applied. The representative sequences, which were generated after denoising were used to assign bacterial taxonomy using a sklearn-based Naive Bayes classifier trained on the SILVA v138 99% 16S full-length database. The Relative classification frequency table represented differential abundance tests at specific taxonomic levels was created using collapse and feature-table within the QIIME2 plugins (Supplementary Table S2). The "diversity" QIIME2 plugin was used to estimate alpha-diversity measurements and plots using R bioinformatics packages. This microbial diversity analysis pipeline was designed to use the ASVs table (a higher-resolution analog than the traditional OTU table) of the ASVs picking step as necessary input data. Analyzing the differences in species richness and evenness scores considering with the sampling depth was measured using the Observed_OTUs, Chao1, Shannon, Simpson, and Pielou_e alpha-diversity indices (Supplementary Table S3). Each index estimate of both sequencing methods was compared on the basis of different 16S rRNA gene length (V3–V4 vs. 16S full-length) using the Kruskal–Wallis test (a non-parametric version of ANOVA). In addition, a difference of relative abundance between the two methods was analyzed by comparing the average bacterial proportion and composition investigated in each taxonomic ranking. To compare the bacterial classification accuracy of the two methods, we constructed and analyzed a phylogenetic tree generated with sequence inputs for the three bacterial strains (*Bifidobacterium*, *Alistipes*, and *Bacteroides* strains) selected on the classification table at the genus- and species level based on the multiple sequence alignment (MSA) with the SILVA 138v database. The MSA was performed by the IUB DNA weight matrix (transition weight 0.50; delay-divergent cutoff 30%) in the MEGA X genetic analysis software^61^, and the phylogeny trees were illustrated by applying the distance matrix (UPGMA; Neighbor-joining, bootstrap 2000; minimum evolution) based on the MSA results. The bacterial taxon was selected as strains having high sequence similarity between similar species or measuring high proportions on the taxonomy classification table. This tree analysis was performed to determine whether how similarly the sFL16S and V3–V4 taxonomy classifications were to the reference database given the sequence similarity according to the different amplicon regions. Additionally, the bacterial classification accuracy according to the different amplicon regions was cross-checked by comparing the taxonomy matching rate of each ASV taxonomy and NCBI bacterial reference genome database (about 40 K genomes) at the genus and species level. The ASV taxonomy was filtered using the blast-hit tool based on 97% sequence alignment identity (minimum criteria for dividing the species in the microbial genomes). The plot was visualized using the R bioinformatics package.

### Human experiments

Human stool samples were collected from three healthy adults using the NBgen-GUT NP self-collection tube (Noble Biosciences, Republic of Korea). To confirm the significance of the results, a total of 24 samples were prepared by dividing the fecal specimens taken from each participant into eight identical sampling tubes. And then, all collected fecal samples were stored at − 80 °C. The prior informed consents for human experiment were obtained from all subjects before the study began. All participants did not affect microbial communities, such as taking medication from a week before the study began. The human experiment in this study (stool samples collection) was approved by the ethics committee of Theragen Bio (Theragen Bio, Republic of Korea) Internal Review Board (IRB numbers 700062-20180905-JR-005-01). All methods applied to the human experiment of this study were carried out in accordance with the guidelines and regulations of the declaration of Helsinki.

## Supplementary Information

Supplementary Legends.

Supplementary Figure S1.

Supplementary Figure S2.

Supplementary Figure S3.

Supplementary Figure S4.

Supplementary Table S1.

Supplementary Table S2.

Supplementary Table S3.

Supplementary Table S4.

Supplementary Table S5.

## References

[1] Shin J (2016). Analysis of the mouse gut microbiome using full-length 16S rRNA amplicon sequencing. Sci. Rep..

[2] Nash AK (2017). The gut mycobiome of the Human Microbiome Project healthy cohort. Microbiome.

[3] Moffatt MF, Cookson WO (2017). The lung microbiome in health and disease. Clin. Med. (Lond.).

[4] Lloyd-Price J (2017). Strains, functions and dynamics in the expanded Human Microbiome Project. Nature.

[5] D'Argenio V, Salvatore F (2015). The role of the gut microbiome in the healthy adult status. Clin. Chim. Acta.

[6] Cenit MC, Matzaraki V, Tigchelaar EF, Zhernakova A (1842). Rapidly expanding knowledge on the role of the gut microbiome in health and disease. Biochim. Biophys. Acta.

[7] Schloss PD, Handelsman J (2005). Metagenomics for studying unculturable microorganisms: cutting the Gordian knot. Genome Biol..

[8] Kumar PS, Brooker MR, Dowd SE, Camerlengo T (2011). Target region selection is a critical determinant of community fingerprints generated by 16S pyrosequencing. PLoS ONE.

[9] Jiang B, Song K, Ren J, Deng M, Sun F, Zhang X (2012). Comparison of metagenomic samples using sequence signatures. BMC Genom..

[10] Chakravorty S, Helb D, Burday M, Connell N, Alland D (2007). A detailed analysis of 16S ribosomal RNA gene segments for the diagnosis of pathogenic bacteria. J. Microbiol. Methods.

[11] Petrosino JF, Highlander S, Luna RA, Gibbs RA, Versalovic J (2009). Metagenomic pyrosequencing and microbial identification. Clin.. Chem..

[12] Sperling JL (2017). Comparison of bacterial 16S rRNA variable regions for microbiome surveys of ticks. Ticks Tick Borne Dis..

[13] Barb JJ (2016). Development of an analysis pipeline characterizing multiple hypervariable regions of 16S rRNA using mock samples. PLoS ONE.

[14] Walters W (2016). Improved bacterial 16S rRNA gene (V4 and V4–5) and fungal internal transcribed spacer marker gene primers for microbial community surveys. mSystems.

[15] Fadrosh DW (2014). An improved dual-indexing approach for multiplexed 16S rRNA gene sequencing on the Illumina MiSeq platform. Microbiome.

[16] Wagner J (2016). Evaluation of PacBio sequencing for full-length bacterial 16S rRNA gene classification. BMC Microbiol..

[17] Nilsson RH (2019). Mycobiome diversity: high-throughput sequencing and identification of fungi. Nat. Rev. Microbiol..

[18] Wurzbacher C (2019). Introducing ribosomal tandem repeat barcoding for fungi. Mol. Ecol. Resour..

[19] Frank JA (2016). Improved metagenome assemblies and taxonomic binning using long-read circular consensus sequence data. Sci. Rep..

[20] Franzen O (2015). Erratum to: Improved OTU-picking using long-read 16S rRNA gene amplicon sequencing and generic hierarchical clustering. Microbiome.

[21] Jain M (2015). Improved data analysis for the MinION nanopore sequencer. Nat. Methods.

[22] Au KF, Underwood JG, Lee L, Wong WH (2012). Improving PacBio long read accuracy by short read alignment. PLoS ONE.

[23] Ardui S, Ameur A, Vermeesch JR, Hestand MS (2018). Single molecule real-time (SMRT) sequencing comes of age: applications and utilities for medical diagnostics. Nucleic Acids Res..

[24] Manzari C (2020). Accurate quantification of bacterial abundance in metagenomic DNAs accounting for variable DNA integrity levels. Microb Genom..

[25] Klemetsen T, Willassen NP, Karlsen CR (2019). Full-length 16S rRNA gene classification of Atlantic salmon bacteria and effects of using different 16S variable regions on community structure analysis. Microbiologyopen.

[26] The Human Microbiome Project Consortium (2012). Structure, function and diversity of the healthy human microbiome. Nature.

[27] Mariat D (2009). The Firmicutes/Bacteroidetes ratio of the human microbiota changes with age. BMC Microbiol..

[28] Qin J (2010). A human gut microbial gene catalogue established by metagenomic sequencing. Nature.

[29] Ansaldo E (2019). *Akkermansia muciniphila* induces intestinal adaptive immune responses during homeostasis. Science.

[30] Kasahara K (2018). Interactions between *Roseburia intestinalis* and diet modulate atherogenesis in a murine model. Nat. Microbiol..

[31] Miquel S (2013). *Faecalibacterium prausnitzii* and human intestinal health. Curr. Opin. Microbiol..

[32] Ogata Y (2019). Complete genome sequence of *Phascolarctobacterium faecium* JCM 30894, a succinate-utilizing bacterium isolated from human feces. Microbiol. Resour. Announc..

[33] Ohkusa T (2002). *Fusobacterium varium* localized in the colonic mucosa of patients with ulcerative colitis stimulates species-specific antibody. J. Gastroenterol. Hepatol..

[34] Lugli GA (2018). Tracking the taxonomy of the genus bifidobacterium based on a phylogenomic approach. Appl. Environ. Microbiol..

[35] Mianzhi Y, Shah NP (2017). Contemporary nucleic acid-based molecular techniques for detection, identification, and characterization of Bifidobacterium. Crit. Rev. Food Sci. Nutr..

[36] Tannock GW (1999). Identification of lactobacilli and bifidobacteria. Curr. Issues Mol. Biol..

[37] Youn SY, Seo JM, Ji GE (2008). Evaluation of the PCR method for identification of Bifidobacterium species. Lett. Appl. Microbiol..

[38] Ehrlich, S. D. in *Metagenomics of the Human Body* Ch. Chapter 15, 307–316 (2011).

[39] Gevers D (2012). The Human Microbiome Project: a community resource for the healthy human microbiome. PLoS Biol..

[40] Heiman ML, Greenway FL (2016). A healthy gastrointestinal microbiome is dependent on dietary diversity. Mol. Metab..

[41] Halfvarson J (2017). Dynamics of the human gut microbiome in inflammatory bowel disease. Nat. Microbiol..

[42] Sun MF, Shen YQ (2018). Dysbiosis of gut microbiota and microbial metabolites in Parkinson's Disease. Ageing Res. Rev..

[43] Tomasello G (2016). Nutrition, oxidative stress and intestinal dysbiosis: Influence of diet on gut microbiota in inflammatory bowel diseases. Biomed. Pap. Med. Fac. Univ. Palacky. Olomouc Czech Repub...

[44] Rapin A, Pattaroni C, Marsland BJ, Harris NL (2017). Microbiota analysis using an Illumina MiSeq platform to sequence 16S rRNA genes. Curr. Protoc. Mouse Biol..

[45] Yarza P (2014). Uniting the classification of cultured and uncultured bacteria and archaea using 16S rRNA gene sequences. Nat. Rev. Microbiol..

[46] Gupta A, Sharma VK (2015). Using the taxon-specific genes for the taxonomic classification of bacterial genomes. BMC Genom..

[47] McIntyre ABR (2017). Comprehensive benchmarking and ensemble approaches for metagenomic classifiers. Genome Biol..

[48] Ye SH, Siddle KJ, Park DJ, Sabeti PC (2019). Benchmarking metagenomics tools for taxonomic classification. Cell.

[49] Klindworth A (2013). Evaluation of general 16S ribosomal RNA gene PCR primers for classical and next-generation sequencing-based diversity studies. Nucleic Acids Res..

[50] Fuks G (2018). Combining 16S rRNA gene variable regions enables high-resolution microbial community profiling. Microbiome.

[51] Johnson JS (2019). Evaluation of 16S rRNA gene sequencing for species and strain-level microbiome analysis. Nat. Commun..

[52] Ravi RK, Walton K, Khosroheidari M (2018). MiSeq: a next generation sequencing platform for genomic analysis. Methods Mol. Biol..

[53] Rossi-Tamisier M, Benamar S, Raoult D, Fournier PE (2015). Cautionary tale of using 16S rRNA gene sequence similarity values in identification of human-associated bacterial species. Int. J. Syst. Evol. Microbiol..

[54] Yuan C, Lei J, Cole J, Sun Y (2015). Reconstructing 16S rRNA genes in metagenomic data. Bioinformatics.

[55] Callahan BJ (2019). High-throughput amplicon sequencing of the full-length 16S rRNA gene with single-nucleotide resolution. Nucleic Acids Res..

[56] Myer PR, Kim M, Freetly HC, Smith TP (2016). Metagenomic and near full-length 16S rRNA sequence data in support of the phylogenetic analysis of the rumen bacterial community in steers. Data Brief.

[57] Zhang J (2018). Evaluation of different 16S rRNA gene V regions for exploring bacterial diversity in a eutrophic freshwater lake. Sci. Total Environ..

[58] Wallis KF, Melnyk SB, Miousse IR (2020). Sex-specific effects of dietary methionine restriction on the intestinal microbiome. Nutrients.

[59] Callahan BJ (2016). DADA2: High-resolution sample inference from Illumina amplicon data. Nat. Methods.

[60] Park C (2020). Performance comparison of fecal preservative and stock solutions for gut microbiome storage at room temperature. J. Microbiol..

[61] Ryan SM (2020). Evolutionarily conserved transcription factors drive the oxidative stress response in Drosophila. J. Exp. Biol..

