# Publisher Correction: The effect of taxonomic classification by full-length 16S rRNA sequencing with a synthetic long-read technology

**DOI:** 10.1038/s41598-021-90067-z

**Published:** 2021-05-19

**Authors:** Jinuk Jeong, Kyeongeui Yun, Seyoung Mun, Won‑Hyong Chung, Song‑Yi Choi, Young‑do Nam, Mi Young Lim, Chang Pyo Hong, ChanHyeok Park, Yong Ju Ahn, Kyudong Han

**Affiliations:** 1 Department of Nanobiomedical Science, Dankook University, Cheonan, 31116 Republic of Korea; 2 Microbiome Division, Theragen Bio Co., Ltd, Seongnam‑si, Gyeonggi‑do 13488 Republic of Korea; 3 Center for Bio-Medical Engineering Core Facility, Dankook University, Cheonan, 31116 Republic of Korea; 4 Research Group of Healthcare, Korea Food Research Institute, Wanju, 55365 Republic of Korea; 5 Department of Pathology, School of Medicine, Chungnam National University, Daejeon, 35015 Republic of Korea; 6 Department of Microbiology, College of Science and Technology, Dankook University, Cheonan, 31116 Republic of Korea; 7 Department of Food Biotechnology, Korea University of Science and Technology, Daejeon, 34113 Republic of Korea

Correction to: *Scientific Reports* 10.1038/s41598-020-80826-9, published online 18 January 2021

The original version of this Article contained a typographical error in the spelling of the author Yong Ju Ahn, which was incorrectly given as Yong Ahn. This has now been corrected in the PDF and HTML versions of the Article.

