# Supplementary material for: The effect of taxonomic classification by full-length 16S rRNA sequencing with a synthetic long-read technology
Source: Sci Rep. 2021 Jan 18;11:1727. doi: 10.1038/s41598-020-80826-9 (PMC7814050; doi:10.1038/s41598-020-80826-9)
Supplement: Supplementary file 1 — Supplementary Legends. [file 41598_2020_80826_MOESM1_ESM.docx]

**The effect of taxonomic classification by full-length 16S rRNA sequencing with a synthetic long-read technology**

**Jinuk Jeong^1,‡^, Kyeongeui Yun^2,‡^, Seyoung Mun^1,3,^, Won-Hyong Chung^4^ , Song-Yi Choi^5^, Young-do Nam^4,7^, Mi Young Lim^4^, Chang Pyo Hong^2^, ChanHyeok Park^2^, Yong Ju Ahn^2,*^ and Kyudong Han^3,6,*^**

^1^ Department of Nanobiomedical Science, Dankook University, Cheonan 31116, Republic of Korea

^2^ Microbiome Division, Theragen Bio Co., Ltd., Seongnam-si, Gyeonggi-do 13488, Republic of Korea

^3^ Center for Bio‑Medical Engineering Core Facility, Dankook University, Cheonan 31116, Republic of Korea

^4^ Research Group of Healthcare, Korea Food Research Institute, Wanju 55365, Republic of Korea

^5^ Department of Pathology, School of Medicine, Chungnam National University, Daejeon 35015, Republic of Korea

^6^ Department of Microbiology, College of Science & Technology, Dankook University, Cheonan 31116, Republic of Korea

^7^ Department of Food Biotechnology, Korea University of Science and Technology, Daejeon 34113, Republic of Korea

‡ These authors contributed equally

**Supplementary Data**

Figures S1-S4

Tables S1-S5

**Supplementary Figure legends**

**Supplementary Figure S1. Relative abundance of human GUT microbiota identified from V3V4 and** **sFL16S methods (class, order, and family).**

Relative abundance bar plots represent the bacterial composition in human GUT microbiota at the class, order, and family levels, identified from V3V4 and sFL16S methods, respectively. There was no significant difference in bacterial genus composition and proportions between both methods. Each legend box shows the top 10 classified bacterial taxa among the whole proportion.

**Supplementary Figure S2. Relative abundance of distinct bacterial species particularly identified from V3V4 and sFL16S methods.**

Relative abundance bar plots represent the relative proportions of distinct bacterial taxonomy at the species level particularly identified from two different methods, respectively. There was significant difference in non-specifically defined species (e.g., 'uncultured bacteria' and 'Human gut') proportions between two different methods. Each legend box shows the top 10 classified bacterial taxa among the whole proportion.

**Supplementary Figure S3. Relative abundance of distinct bacterial species identified inter-individually using V3V4 and sFL16S methods.**

Relative abundance bar plots represent the relative proportions of distinct bacterial taxonomy at the species level particularly identified inter-individually using two different methods, respectively. There was significant difference in non-specifically defined species (e.g., 'uncultured bacteria' and 'Human gut') proportions between two different methods. Each legend box shows the top six classified bacterial taxa among the whole proportion.

**Supplementary Figure S4. Phylogenetic tree analysis for *Bacteroides* and *Alistipes sp.***

(a, c) Neighbor-joining phylogenetic tree analysis of bacterial strains belonging to the two genera (*Bacteroides* and *Alistipes*). The reference sequence was obtained from SILVA 138v DB. (b, d) Multiple sequence alignment (MSA) showing the difference in sequence similarity to the reference database. The MSA results visualized using the NCBI Multiple Sequence Alignment Viewer 1.16.1v. The sFL16S ASV sequences are substantially identical to the reference database compared to the V3V4 ASVs and were defined as the exact bacterial taxon at the species level.

**Supplementary Table lists**

**Supplementary Table S1. Statistics of metagenome sequencing data.**

**Supplementary Table S2. Taxonomic classification results for feature ID with more 70% (default) confidence threshold.**

**Supplementary Table S3. Quantified alpha-diversity statistics of each sample.**

**Supplementary Table S4. Relative abundance of each classified bacterial taxon.**

**Supplementary Table S5. Taxonomy matching rate of bacterial ASVs with NCBI database.**
