# Supplementary figures and images for: The effect of taxonomic classification by full-length 16S rRNA sequencing with a synthetic long-read technology
Source: Sci Rep. 2021 Jan 18;11:1727. doi: 10.1038/s41598-020-80826-9 (PMC7814050; doi:10.1038/s41598-020-80826-9)

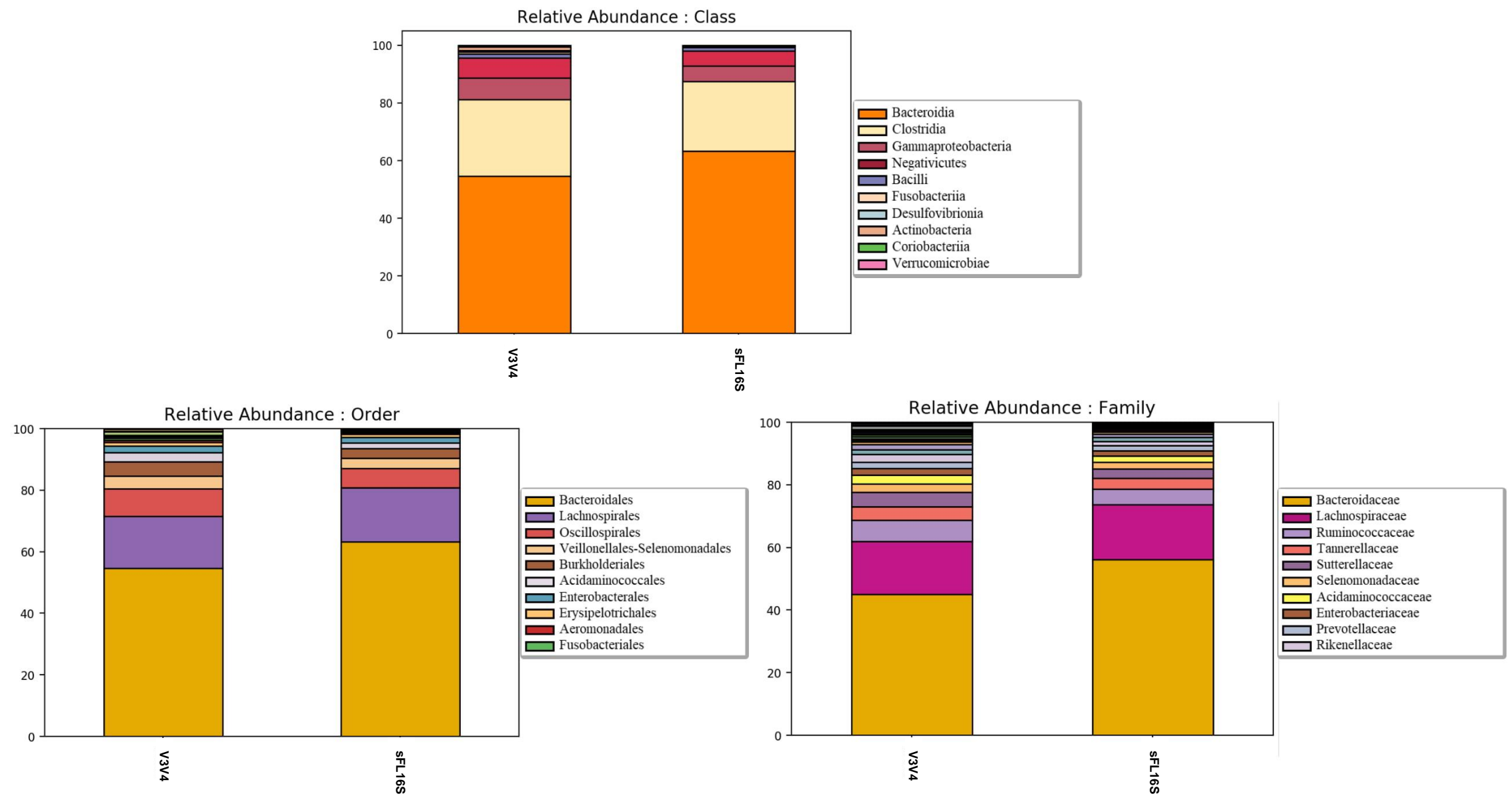

Supplementary Figure S1.

Supplement: Supplementary file 2 — Supplementary Figure S1. [file 41598_2020_80826_MOESM2_ESM.pdf]

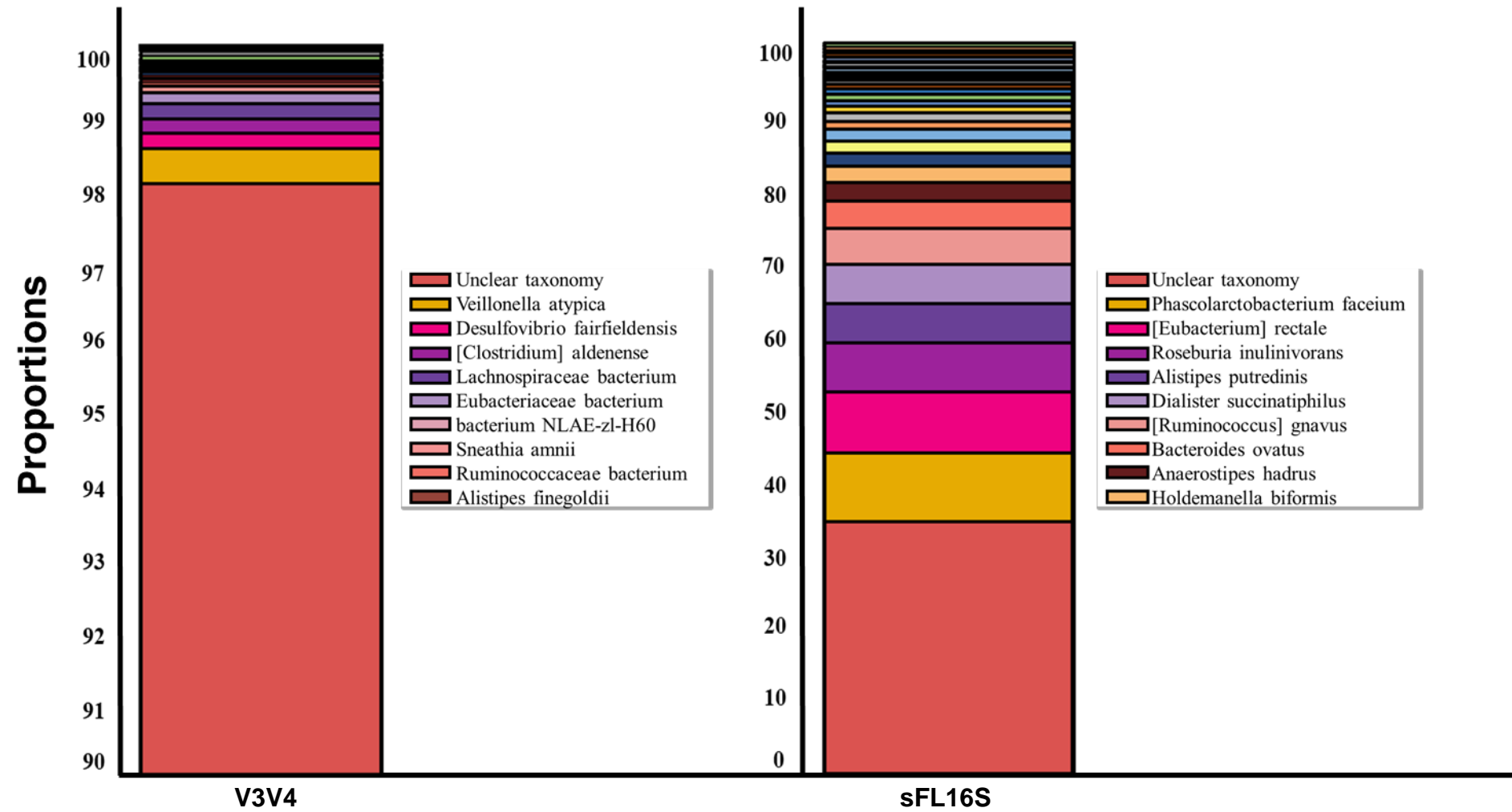

Supplementary Figure S2.

Supplement: Supplementary file 3 — Supplementary Figure S2. [file 41598_2020_80826_MOESM3_ESM.pdf]

Proportions

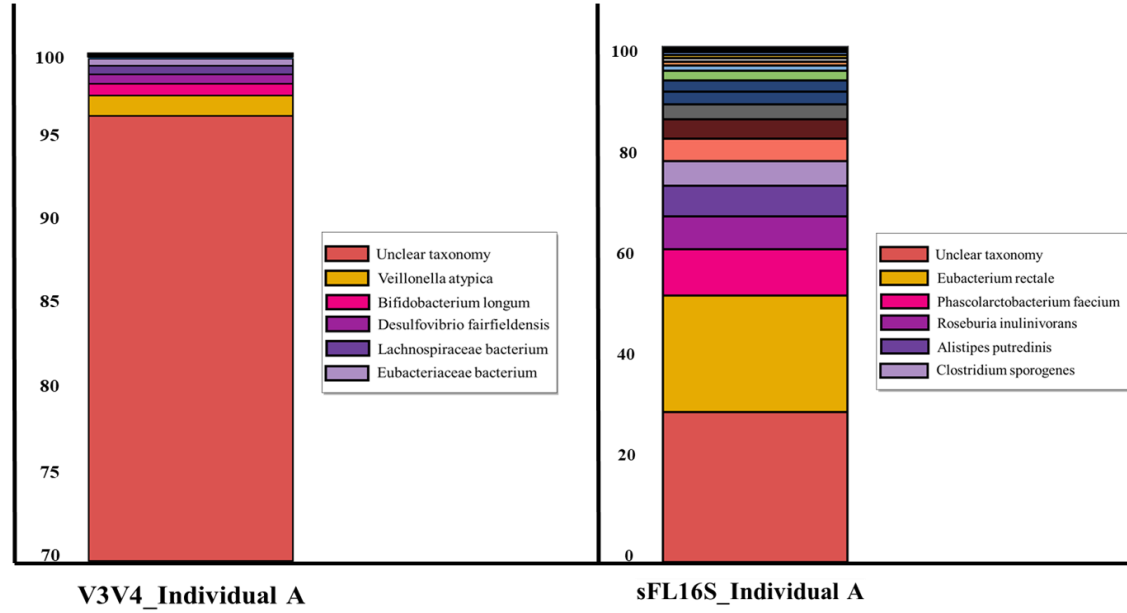

Proportions

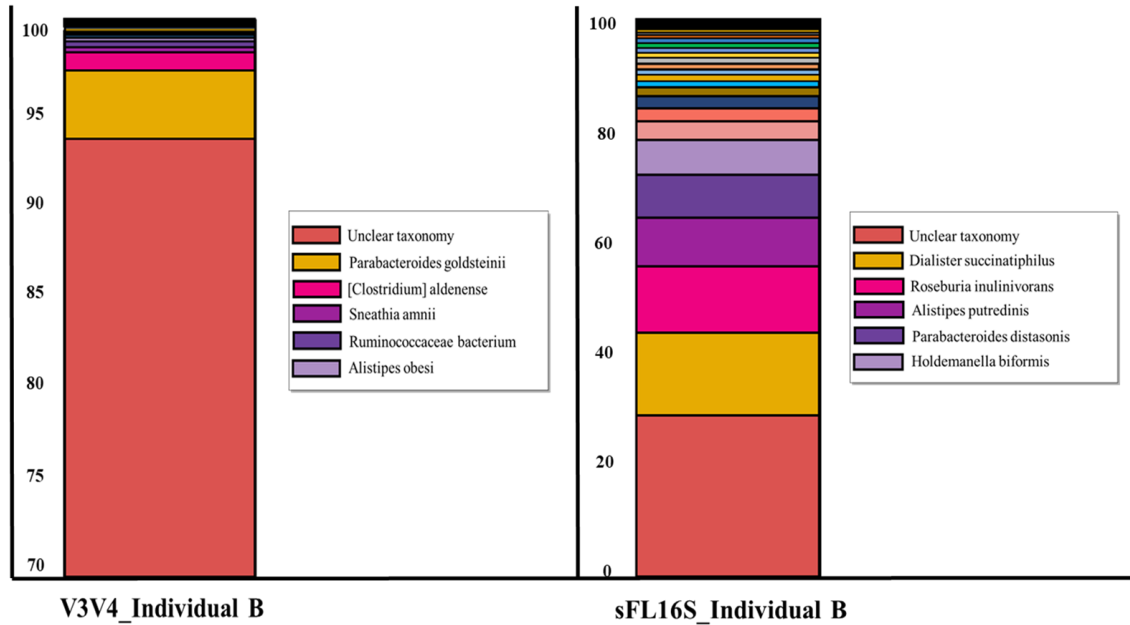

Proportions

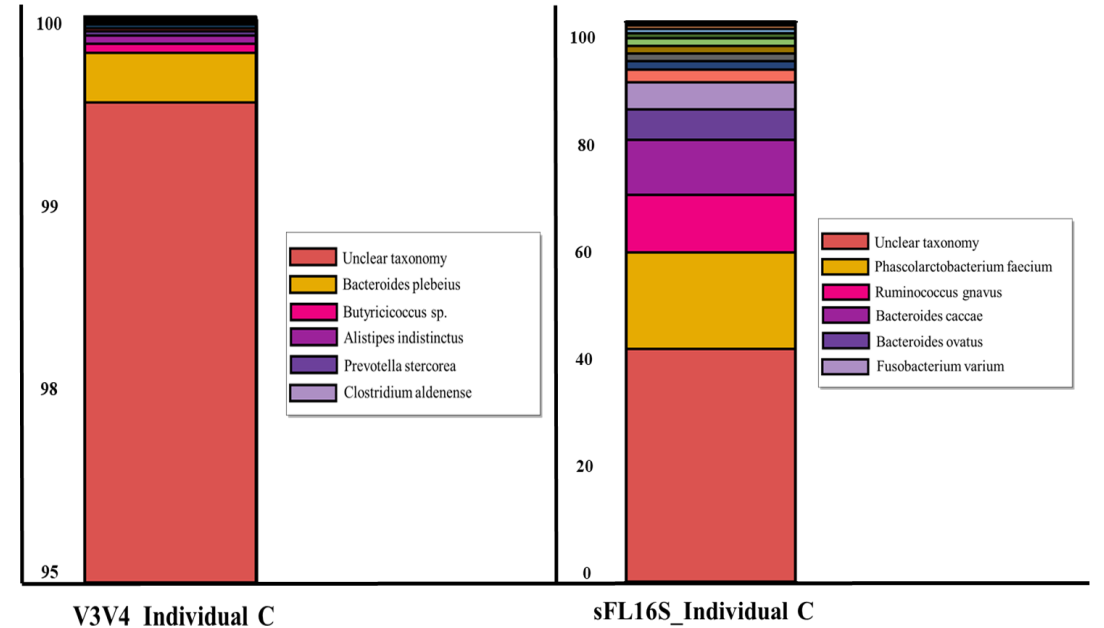

Supplementary Figure S3.

Supplement: Supplementary file 4 — Supplementary Figure S3. [file 41598_2020_80826_MOESM4_ESM.pdf]
