# Supplementary material for: The effect of taxonomic classification by full-length 16S rRNA sequencing with a synthetic long-read technology
Source: Sci Rep. 2021 Jan 18;11:1727. doi: 10.1038/s41598-020-80826-9 (PMC7814050; doi:10.1038/s41598-020-80826-9)
Supplement: Supplementary file 5 — Supplementary Figure S4. [file 41598_2020_80826_MOESM5_ESM.pdf]

a

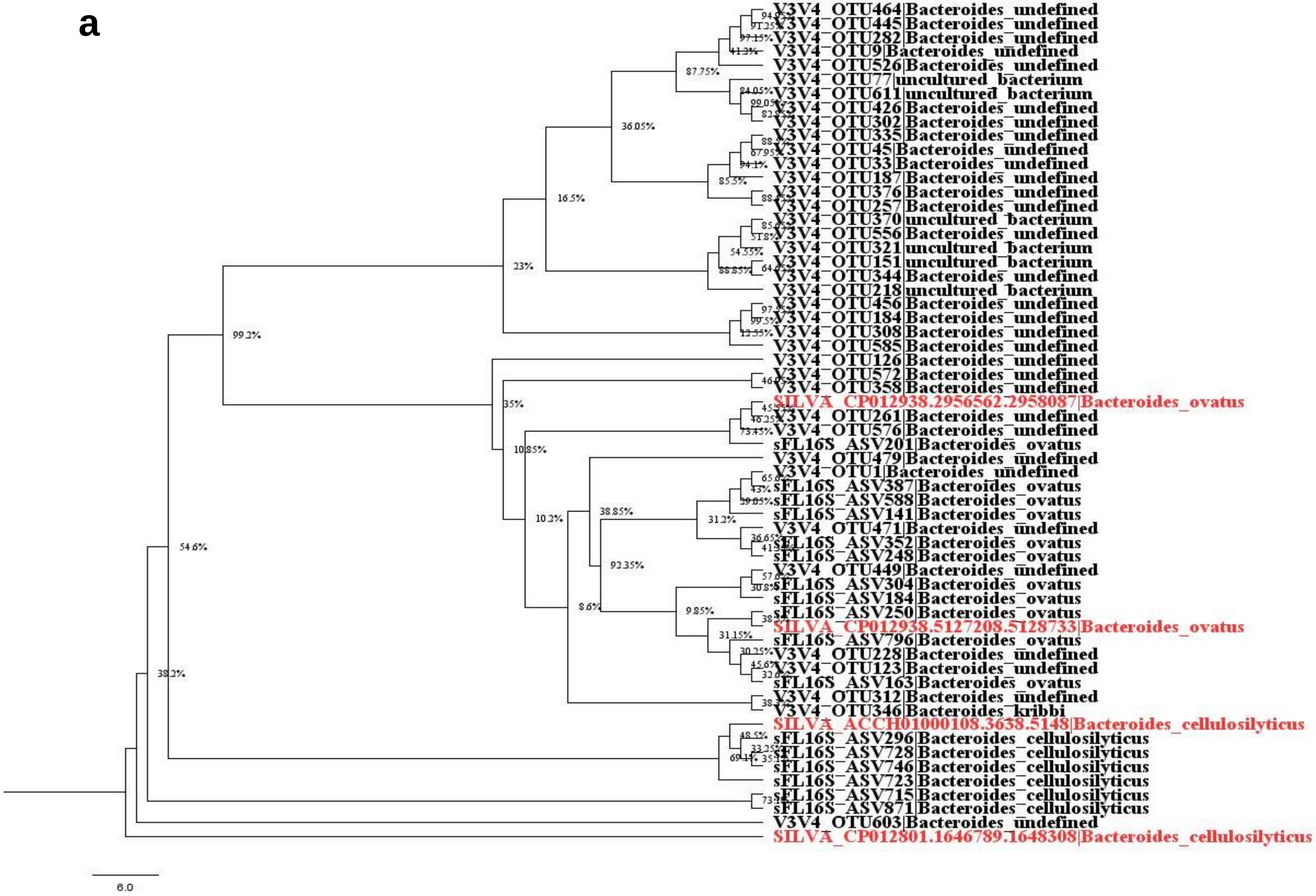
V3V4_OTU464|Bacteroides_undefined
V3V4_OTU445|Bacteroides_undefined
V3V4_OTU282|Bacteroides_undefined
V3V4_OTU9|Bacteroides_undefined
V3V4_OTU526|Bacteroides_undefined
V3V4_OTU77|uncultured_bacterium
V3V4_OTU611|uncultured_bacterium
V3V4_OTU426|Bacteroides_undefined
V3V4_OTU302|Bacteroides_undefined
V3V4_OTU335|Bacteroides_undefined
V3V4_OTU45|Bacteroides_undefined
V3V4_OTU33|Bacteroides_undefined
V3V4_OTU187|Bacteroides_undefined
V3V4_OTU376|Bacteroides_undefined
V3V4_OTU257|Bacteroides_undefined
V3V4_OTU370|uncultured_bacterium
V3V4_OTU556|Bacteroides_undefined
V3V4_OTU321|uncultured_bacterium
V3V4_OTU151|uncultured_bacterium
V3V4_OTU344|Bacteroides_undefined
V3V4_OTU218|uncultured_bacterium
V3V4_OTU456|Bacteroides_undefined
V3V4_OTU184|Bacteroides_undefined
V3V4_OTU308|Bacteroides_undefined
V3V4_OTU585|Bacteroides_undefined
V3V4_OTU126|Bacteroides_undefined
V3V4_OTU572|Bacteroides_undefined
V3V4_OTU358|Bacteroides_undefined
SILVA_CP012938.2956562.2958087|Bacteroides_ovatus
V3V4_OTU261|Bacteroides_undefined
V3V4_OTU576|Bacteroides_undefined
sFL16S_ASV201|Bacteroides_ovatus
V3V4_OTU479|Bacteroides_undefined
V3V4_OTU1|Bacteroides_undefined
sFL16S_ASV387|Bacteroides_ovatus
sFL16S_ASV588|Bacteroides_ovatus
sFL16S_ASV141|Bacteroides_ovatus
V3V4_OTU471|Bacteroides_undefined
sFL16S_ASV352|Bacteroides_ovatus
sFL16S_ASV248|Bacteroides_ovatus
V3V4_OTU449|Bacteroides_undefined
sFL16S_ASV304|Bacteroides_ovatus
sFL16S_ASV184|Bacteroides_ovatus
sFL16S_ASV250|Bacteroides_ovatus
SILVA_CP012938.5127208.5128733|Bacteroides_ovatus
sFL16S_ASV796|Bacteroides_ovatus
V3V4_OTU228|Bacteroides_undefined
V3V4_OTU123|Bacteroides_undefined
sFL16S_ASV163|Bacteroides_ovatus
V3V4_OTU312|Bacteroides_undefined
V3V4_OTU346|Bacteroides_kribbi
SILVA_ACCH01000108.3638.5148|Bacteroides_cellulosilyticus
sFL16S_ASV296|Bacteroides_cellulosilyticus
sFL16S_ASV728|Bacteroides_cellulosilyticus
sFL16S_ASV746|Bacteroides_cellulosilyticus
sFL16S_ASV723|Bacteroides_cellulosilyticus
sFL16S_ASV715|Bacteroides_cellulosilyticus
sFL16S_ASV871|Bacteroides_cellulosilyticus
V3V4_OTU603|Bacteroides_undefined
SILVA_CP012801.1646789.1648308|Bacteroides_cellulosilyticus
6.0

b

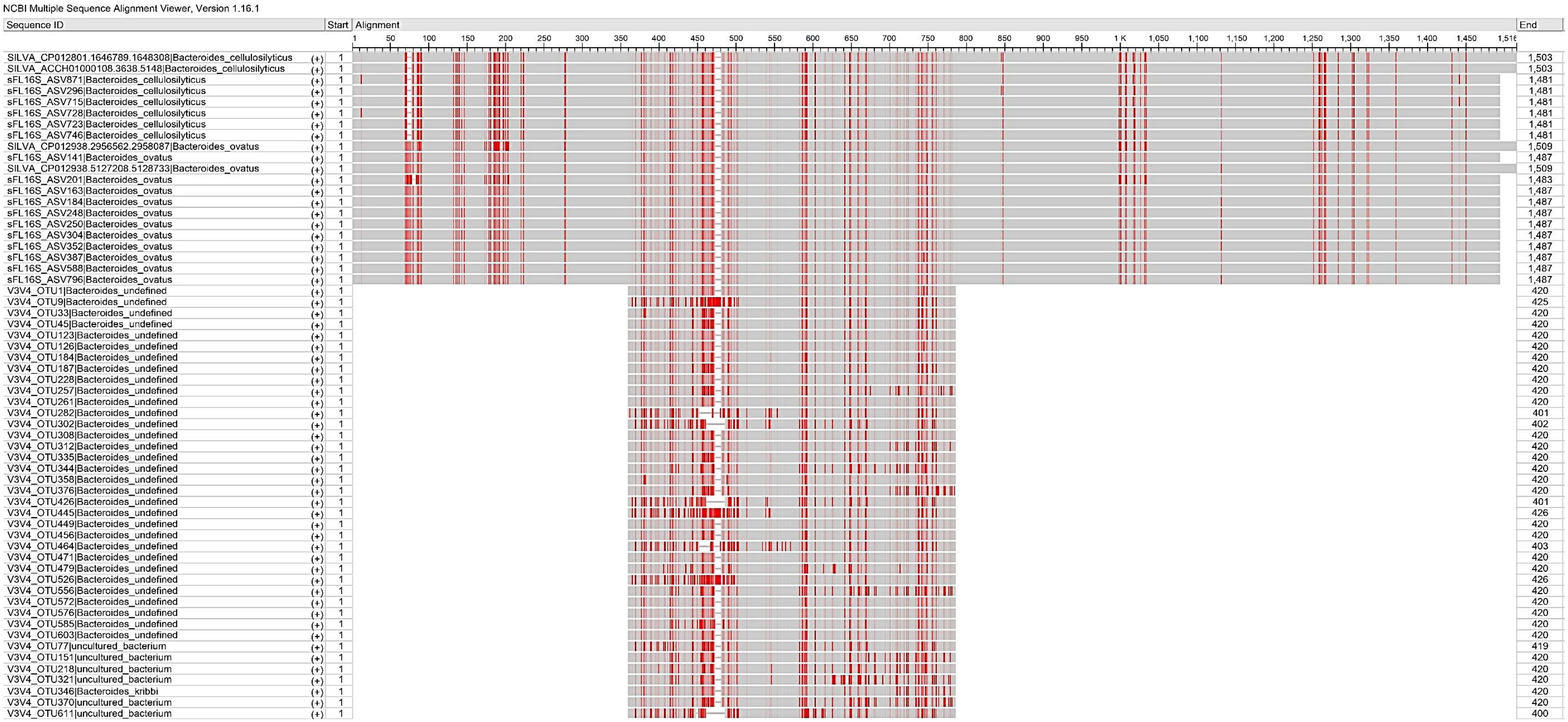
NCBI Multiple Sequence Alignment Viewer, Version 1.16.1
Sequence ID
Start
Alignment
End
1 50 100 150 200 250 300 350 400 450 500 550 600 650 700 750 800 850 900 950 1 K 1,050 1,100 1,150 1,200 1,250 1,300 1,350 1,400 1,450 1,516
SILVA_CP012801.1646789.1648308|Bacteroides_cellulosilyticus (+) 1 1,503
SILVA_ACCH01000108.3638.5148|Bacteroides_cellulosilyticus (+) 1 1,503
sFL16S_ASV871|Bacteroides_cellulosilyticus (+) 1 1,481
sFL16S_ASV296|Bacteroides_cellulosilyticus (+) 1 1,481
sFL16S_ASV715|Bacteroides_cellulosilyticus (+) 1 1,481
sFL16S_ASV728|Bacteroides_cellulosilyticus (+) 1 1,481
sFL16S_ASV723|Bacteroides_cellulosilyticus (+) 1 1,481
sFL16S_ASV746|Bacteroides_cellulosilyticus (+) 1 1,481
SILVA_CP012938.2956562.2958087|Bacteroides_ovatus (+) 1 1,509
sFL16S_ASV141|Bacteroides_ovatus (+) 1 1,487
SILVA_CP012938.5127208.5128733|Bacteroides_ovatus (+) 1 1,509
sFL16S_ASV201|Bacteroides_ovatus (+) 1 1,483
sFL16S_ASV163|Bacteroides_ovatus (+) 1 1,487
sFL16S_ASV184|Bacteroides_ovatus (+) 1 1,487
sFL16S_ASV248|Bacteroides_ovatus (+) 1 1,487
sFL16S_ASV250|Bacteroides_ovatus (+) 1 1,487
sFL16S_ASV304|Bacteroides_ovatus (+) 1 1,487
sFL16S_ASV352|Bacteroides_ovatus (+) 1 1,487
sFL16S_ASV387|Bacteroides_ovatus (+) 1 1,487
sFL16S_ASV588|Bacteroides_ovatus (+) 1 1,487
sFL16S_ASV796|Bacteroides_ovatus (+) 1 1,487
V3V4_OTU1|Bacteroides_undefined (+) 1 420
V3V4_OTU9|Bacteroides_undefined (+) 1 425
V3V4_OTU33|Bacteroides_undefined (+) 1 420
V3V4_OTU45|Bacteroides_undefined (+) 1 420
V3V4_OTU123|Bacteroides_undefined (+) 1 420
V3V4_OTU126|Bacteroides_undefined (+) 1 420
V3V4_OTU184|Bacteroides_undefined (+) 1 420
V3V4_OTU187|Bacteroides_undefined (+) 1 420
V3V4_OTU228|Bacteroides_undefined (+) 1 420
V3V4_OTU257|Bacteroides_undefined (+) 1 420
V3V4_OTU261|Bacteroides_undefined (+) 1 420
V3V4_OTU282|Bacteroides_undefined (+) 1 401
V3V4_OTU302|Bacteroides_undefined (+) 1 402
V3V4_OTU308|Bacteroides_undefined (+) 1 420
V3V4_OTU312|Bacteroides_undefined (+) 1 420
V3V4_OTU335|Bacteroides_undefined (+) 1 420
V3V4_OTU344|Bacteroides_undefined (+) 1 420
V3V4_OTU358|Bacteroides_undefined (+) 1 420
V3V4_OTU376|Bacteroides_undefined (+) 1 420
V3V4_OTU426|Bacteroides_undefined (+) 1 401
V3V4_OTU445|Bacteroides_undefined (+) 1 426
V3V4_OTU449|Bacteroides_undefined (+) 1 420
V3V4_OTU456|Bacteroides_undefined (+) 1 420
V3V4_OTU464|Bacteroides_undefined (+) 1 403
V3V4_OTU471|Bacteroides_undefined (+) 1 420
V3V4_OTU479|Bacteroides_undefined (+) 1 420
V3V4_OTU526|Bacteroides_undefined (+) 1 426
V3V4_OTU556|Bacteroides_undefined (+) 1 420
V3V4_OTU572|Bacteroides_undefined (+) 1 420
V3V4_OTU576|Bacteroides_undefined (+) 1 420
V3V4_OTU585|Bacteroides_undefined (+) 1 420
V3V4_OTU603|Bacteroides_undefined (+) 1 420
V3V4_OTU77|uncultured_bacterium (+) 1 419
V3V4_OTU151|uncultured_bacterium (+) 1 420
V3V4_OTU218|uncultured_bacterium (+) 1 420
V3V4_OTU321|uncultured_bacterium (+) 1 420
V3V4_OTU346|Bacteroides_kribbi (+) 1 420
V3V4_OTU370|uncultured_bacterium (+) 1 420
V3V4_OTU611|uncultured_bacterium (+) 1 400

c

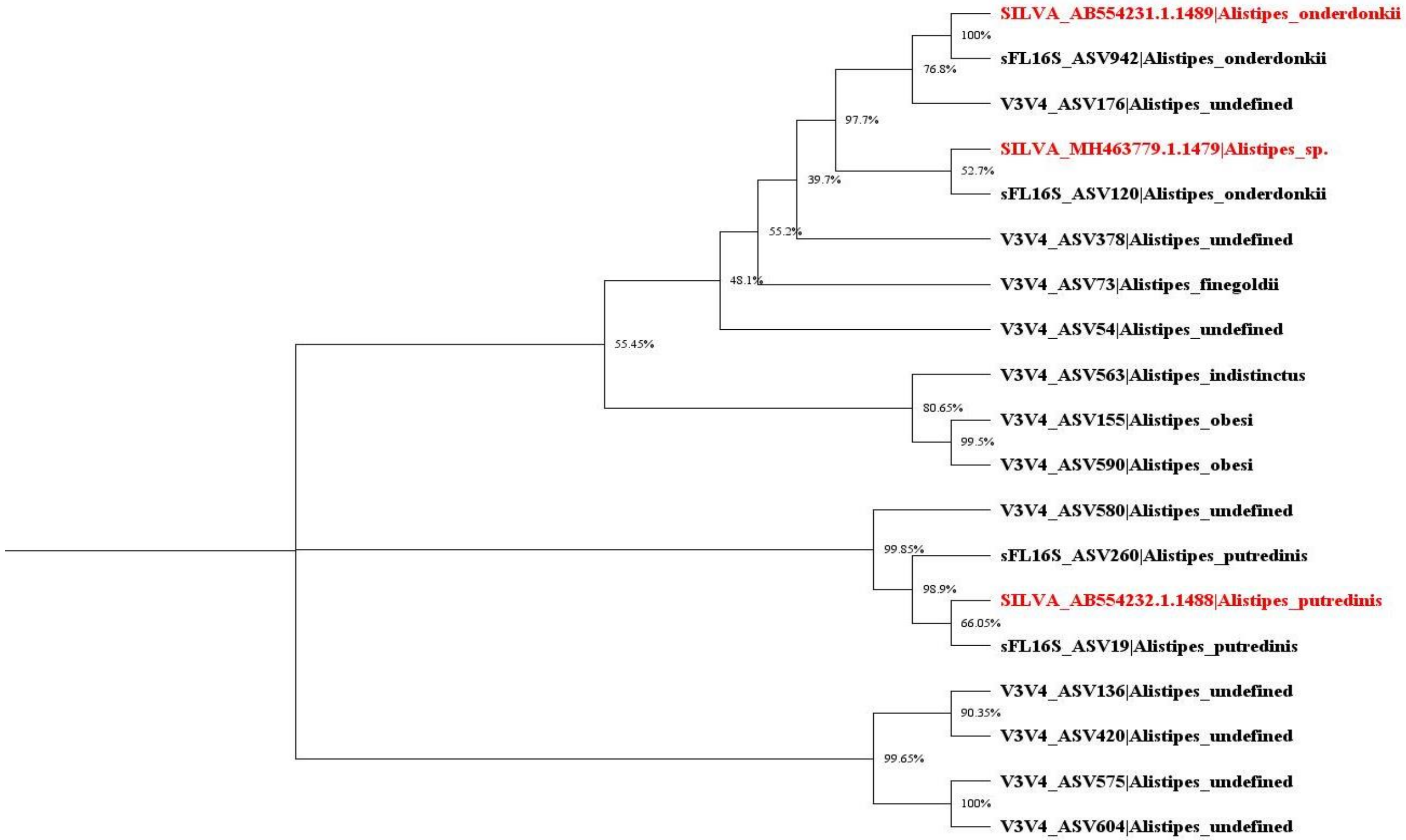

d

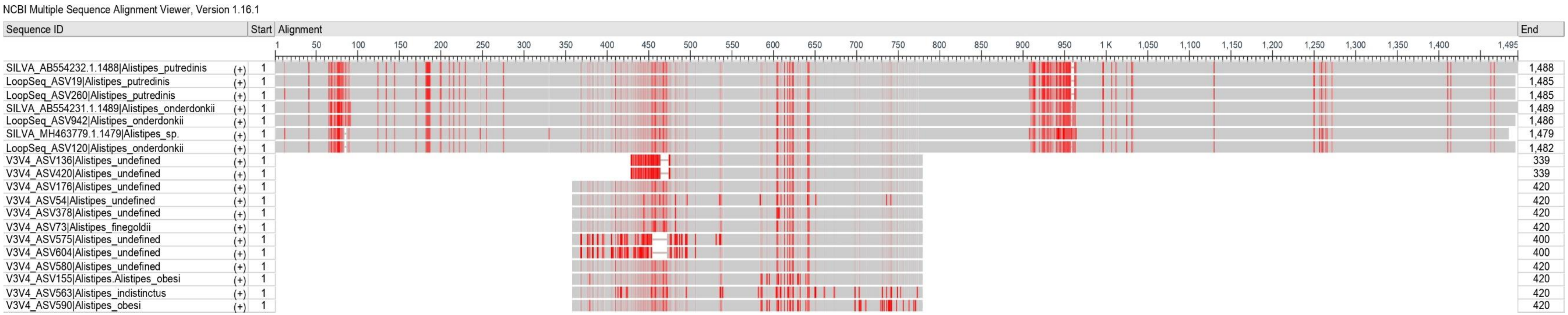

Supplementary Figure S4.
